# Associations of SEMA7A, SEMA4D, ADAMTS10, and ADAM8 with KRAS, NRAS, BRAF, PIK3CA, and AKT Gene Mutations, Microsatellite Instability Status, and Cytokine Expression in Colorectal Cancer Tissue

**DOI:** 10.3390/cimb46090609

**Published:** 2024-09-15

**Authors:** Błażej Ochman, Piotr Limanówka, Sylwia Mielcarska, Agnieszka Kula, Miriam Dawidowicz, Wiktor Wagner, Dorota Hudy, Monika Szrot, Jerzy Zbigniew Piecuch, Jerzy Piecuch, Zenon Czuba, Elżbieta Świętochowska

**Affiliations:** 1 Department of Medical and Molecular Biology, Faculty of Medical Sciences in Zabrze, Medical University of Silesia, 19 Jordana, 41-808 Zabrze, Poland; d201228@365.sum.edu.pl (B.O.); s82955@365.sum.edu.pl (P.L.); d201109@365.sum.edu.pl (S.M.); s86294@365.sum.edu.pl (W.W.); dorota.hudy@sum.edu.pl (D.H.); 2 Department of Oncological Surgery, Faculty of Medical Sciences in Zabrze, Medical University of Silesia, 41-808 Katowice, Poland; d201070@365.sum.edu.pl (A.K.); d201069@365.sum.edu.pl (M.D.); 3 Department of General and Bariatric Surgery and Emergency Medicine in Zabrze, Faculty of Medical Sciences in Zabrze, Medical University of Silesia, 10 Marii Curie-Skłodowskiej, 41-800 Zabrze, Poland; mszrot@sum.edu.pl (M.S.); jpiecuch@sum.edu.pl (J.P.); 4 Department of Microbiology and Immunology, Faculty of Medical Sciences in Zabrze, Medical University of Silesia, 19 Jordana, 41-808 Zabrze, Poland; zczuba@sum.edu.pl

**Keywords:** semaphorin 7A (SEMA7A), ADAM (a disintegrin and metalloprotease) proteins, ADAMTS10, ADAM8, SEMA4D, colorectal cancer (CRC), microenvironment, tumor, K-ras oncogene, proto-oncogene proteins B-raf (BRAF), instability, microsatellite

## Abstract

Semaphorins (SEMAs), ADAM, and ADAMTS family members are implicated in various cancer progression events within the tumor microenvironment across different cancers. In this study, we aimed to evaluate the expression of SEMA7A, SEMA4D, ADAM8, and ADAMTS10 in colorectal cancer (CRC) in relation to the mutational landscape of KRAS, NRAS, BRAF, PIK3CA, and AKT genes, microsatellite instability (MSI) status, and clinicopathological features. We also examined the associations between the expression of these proteins and selected cytokines, chemokines, and growth factors, assessed using a multiplex assay. Protein concentrations were quantified using ELISA in CRC tumors and tumor-free surgical margin tissue homogenates. Gene mutations were evaluated via RT-PCR, and MSI status was determined using immunohistochemistry (IHC). GSEA and statistical analyses were performed using R Studio. We observed a significantly elevated expression of SEMA7A in BRAF-mutant CRC tumors and an overexpression of ADAM8 in KRAS 12/13-mutant tumors. The expression of ADAMTS10 was decreased in PIK3CA-mutant CRC tumors. No significant differences in the expression of the examined proteins were observed based on MSI status. The SEMA7A and SEMA4D expressions were correlated with the expression of numerous cytokines associated with various immune processes. The potential immunomodulatory functions of these molecules and their suitability as therapeutic targets require further investigation.

## 1. Introduction

In recent decades, advances in cancer research have led to the intensive development of immunotherapy and other targeted therapies for various solid tumors, including colorectal cancer (CRC). However, certain aspects related to the formation of the immunological properties of the tumor microenvironment (TME) remain insufficiently understood. These aspects include, among others, the yet unexplored impact of molecules with potential immunomodulatory properties on the expression of specific cytokines, growth factors, immune checkpoints, or on the immune cell’s functions, and how interactions among these and other components of the TME are shaped depending on the mutational landscape of the tumor. Understanding these associations remains a challenge, the resolution of which could enhance current immunotherapy or target-therapy methods by identifying potential therapeutic targets that could overcome therapy resistance in specific patient groups, in line with the goals of personalized therapies [1,2].

In recent years, scientific interest has grown regarding the role of particular molecules in CRC tumor progression, notably those belonging to the semaphorin and ADAMTS/ADAM families [3,4]. Semaphorins (SEMAs) are a family of signaling proteins divided into eight subclasses, among which the 22 known vertebrate semaphorins are categorized into subclasses 3–7. SEMA’s signal transduction primarily occurs through their interactions with plexin family receptors, influencing a range of processes that contribute to tumor progression, including the promotion of angiogenesis, lymphangiogenesis, the modulation of immune processes, and direct effects on molecular events within cancer cells. Besides the subclassification of SEMAs, there is also a group known as immunological SEMAs, which includes semaphorin 7A (SEMA7A) and semaphorin 4D (SEMA4D) [5,6].

A disintegrin and Metalloproteinase Domain-Containing Protein 8 (ADAM8) and a disintegrin and Metalloproteinase with Thrombospondin Motifs 10 (ADAMTS10) are molecules belonging to the metzincin protease superfamily [7]. A disintegrin and Metalloproteinase Domain-Containing Proteins (ADAMs) are zinc-dependent proteases. They were shown to regulate the shedding of membrane-bound proteins, growth factors, cytokines, ligands, and receptors. Their structure mainly consists of a prodomain, a metalloprotease region, a disintegrin domain for adhesion, a cysteine-rich region, epidermal-growth-factor (EGF) repeats, and a transmembrane module followed by a cytoplasmic tail. It has been shown that ADAMs are involved in the molecular mechanism of many different diseases, like neurodegenerative diseases and autoimmune diseases. ADAM8 is considered to be an important factor in the development of cancer [8,9]. ADAMTS10 is a member of the a disintegrin and Metalloproteinase with Thrombospondin (ADAMTS) protein group. These are secreted, extracellular enzymes with a similar structure, consisting of a signal peptide at the amino end followed by a variable-length pro-region, a metalloproteinase domain, a disintegrin-like domain, a central repeat motif of the thrombospondin type 1 (TSR) sequence, and a cysteine-rich domain which is followed by a spacer region. Their expression was shown to be connected with carcinogenesis, in particular events such as cell adhesion, migration, proliferation, and angiogenesis. Both ADAM and ADAMTS enzymes are known for their contribution to inflammation. It was shown that they can alter expression of a number of cytokines [9,10].

The associations between the expressions of SEMA7A, SEMA4D, ADAM8, and ADAMTS10 and the mutation status of KRAS, NRAS, BRAF, PIK3CA, and AKT1 genes have not been previously described. Similarly, it is unknown how the expression of these molecules could be influenced by microsatellite instability (MSI) status in CRC. The aim of this study was to determine the concentrations of SEMA7A, SEMA4D, ADAM8, and ADAMTS10 proteins in CRC tissues and surgical margin tissues, and to investigate whether the KRAS, NRAS, BRAF, PIK3CA, and AKT1 gene mutations, as well as MSI status, affect the expression of these proteins. To explore the significance of these molecules in CRC, we also analyzed their expression in relation to various clinical data, such as TNM scale parameters, tumor stage and grade, and other factors. Additionally, the study aimed to examine the relationships between the expression of the studied proteins and groups of selected cytokines, chemokines, and growth factors. The investigation was further enhanced by performing Gene Set Enrichment Analysis (GSEA) for SEMA7A, SEMA4D, ADAM8, and ADAMTS10 in CRC tumors. An assessment of SEMA7A, SEMA4D, ADAM8, and ADAMTS10 expression and their association with aforementioned gene mutations could suggest a therapeutic potential and narrow down clinical factors contributing to the effectiveness of therapy. Investigating the associations between the expression of the studied molecules and the expression of selected cytokine groups may allow for the estimation of the processes relevant to colorectal cancer progression in which these proteins may be involved. This could contribute to a better assessment of the significance of the studied proteins in potential targeted therapies.

## 2. Materials and Methods

### 2.1. Study Group Characteristics

The study group consisted of 87 CRC tumors, while the control group included 87 corresponding surgical margins. The material was collected during CRC surgeries (approval of the Research Ethics Committee PCN/0022/KB1/42/VI/14/16/18/19/20). The study group comprised only tissues with histopathologically confirmed diagnoses of CRC, and in the case of surgical margin tissues, only those without pathological features of CRC were included. The inclusion criteria for the study were as follows: (1) the patient consented to participate in the study, (2) patient aged over 18 years at the time of surgery, and (3) a histopathological confirmation of colorectal adenocarcinoma and tumor-free surgical margin tissue. Failure to meet any of the inclusion criteria resulted in exclusion from the study. The detailed characteristics of the clinicopathological features of the study group are presented in Table 1.

### 2.2. Tissue Homogenization and Enzyme-Linked Immunosorbent Assay (ELISA) Analyses

A total of 87 CRC tumors and their corresponding 87 surgical margin tissues were homogenized according to our previously described homogenization protocol [11]. The CRC tumor tissues and corresponding surgical margins from patients were weighed and homogenized in a phosphate-buffer solution. Homogenization was conducted using a PRO 200 homogenizer (PRO Scientific Inc., Oxford, CT, USA) at a speed of 10,000 rpm. For each tumor tissue and surgical margin, nine volumes of phosphate-buffered saline were added. The resulting suspensions were then sonicated with an ultrasonic cell disrupter (UP100, Hilscher, Germany). The homogenized tissues were centrifuged at 12,000 rpm for 15 min at 4 °C. The total protein concentration was determined by colorimetry using a pyrogallol-red reagent kit (Sentinel Diagnostics, Milano, Italy). The measurements were taken at a wavelength of 600 nm and a temperature of 37 °C using a Technicon RA-XTTM biochemical analyzer (Technicon Instruments Corporation, Mahopac, NY, USA). Afterward, we determined the concentrations of the target proteins using the Enzyme-linked Immunosorbent Assay (ELISA) method. To select the appropriate dilution of the samples in relation to the amount of protein in tissue homogenates and the sensitivity of the used kits, we conducted preliminary assays. After estimating the optimal dilution of tissue homogenates for each of the proteins, we measured the concentrations of SEMA7A, SEMA4D, ADAM8, and ADAMTS10 in both the study and control groups. The measurements were performed using ELISA kits as follows: for SEMA7A with sensitivity ~0.063 ng/mL (SEB447Hu, Cloud Clone, Wuhan, China); for SEMA4D with sensitivity ~0.61 ng/mL (SEB430Hu, Cloud Clone, Wuhan, China) for ADAM8 with sensitivity ~2.52 pg/mL (SEA620Hu, Cloud Clone, Wuhan, China); and for ADAMTS10 with sensitivity ~0.060 ng/mL (SEK210Hu, Cloud Clone, Wuhan, China). The analyses were performed according to the assay protocols described in the instruction manuals for each kit.

### 2.3. Mutational Landscape Evaluation of CRC Tumors

To evaluate the mutational landscape of the CRC tumors included in this study, we performed a mutation analysis of the KRAS, NRAS, BRAF, PIK3CA, and AKT1 genes using the real-time polymerase chain reaction (RT-PCR) method. Genomic DNA was extracted from fresh-frozen CRC tumors stored at −80 °C. The study group included 54 tumor samples. Genomic DNA was isolated using an automated DNA extractor with the commercial Mag-Bind Blood & Tissue DNA HDQ 96 Kit (M6399-00), following the manufacturers protocol. After isolation, the DNA concentration of each sample was quantified via spectrophotometric analysis. According to the RT-PCR kit protocol, we adjusted the DNA concentration of each sample to 2 ng/µL to optimize the input DNA amount for further analysis. RT-PCR was conducted using the CRC-RT48 CRC Mutation Detection Panel for Real-Time PCR—KRAS, NRAS, BRAF, PIK3CA, and AKT1 mutation and cancer (EntroGen, Woodland Hills, LA, USA), following the procedure described in the instruction manual. The utilized PCR kit enabled 48 reactions to detect nucleotide changes in exon 2, 3, and 4 mutations of KRAS, exon 2, 3, and 4 mutations of NRAS, exon 15 mutations of BRAF, exon 9 and 20 mutations of PIK3CA, and the exon 4 mutation of AKT1 (Table 2). The results were read and interpreted using the QuantStudio™ 5 Real-Time PCR System for Human Identification (Thermo Fisher Scientific, Waltham, MA, USA) according to the CRC-PCR kit manufacturer’s instruction, and the QuantStudio 5 system manual. This allowed us to determine the mutational status for KRAS 12/13, KRAS 117, KRAS 61, KRAS 146, KRAS 59, NRAS 12/13, NRAS 117, NRAS 61, NRAS 146, NRAS 59, BRAF 600, PIK3CA 542/545, PIK3CA 1047, and AKT1 E17K in the analyzed CRC tumors.

### 2.4. Microsatellite Instability (MSI) Evaluation

The material for MSI status analysis were representative formalin-fixed, paraffin-embedded (FFPE) CRC tumor tissue blocks. MSI status was assessed using immunohistochemistry (IHC) methods. We analyzed IHC staining for MLH1, MSH2, MSH6, and PMS2. MSI status was evaluated independently by two pathomorphologists. The method has been extensively described in our previous work [12,13,14].

### 2.5. Cytokine Screening Panel and Principal Component Analysis (PCA)

Cytokine, chemokine, and growth factor levels were quantified in 77 CRC tissue homogenates utilizing the Bio-Plex Pro Human Cytokine Screening Panel, 48-Plex (Bio-Rad Laboratories, Hercules, CA, USA), according to the manufacturers protocol from instruction manual. Specifically, a 50 μL portion of CRC tumor homogenate was diluted 1:2 with sample diluent, incubated with antibody-coupled beads, biotinylated secondary antibodies, and then with streptavidin-PE. Standard curves for each molecule were created using the corresponding cytokine standard solutions. Bead measurements were conducted using the Bio-Plex 200 System. Subsequently, the obtained concentration values of the target molecules in the cytokine assay were adjusted by the previously determined total protein levels for the corresponding CRC tumor homogenates. The pertinent molecules whose expression was analyzed in the cytokine screening panel were assigned into groups representing specific processes or signaling pathways, according to the Gene Ontology (GO) terms [15,16] or Kyoto Encyclopedia of Genes and Genomes (KEGG) annotations [17,18] (Table 3). Based on this classification, PCA was conducted. Adjusted to total protein levels, the expression of cytokines, chemokines, and growth factors was normalized via decimal log transformation and subjected to PCA. To perform PCA, we calculated the covariance matrix of the scaled cytokine expression data, performed eigenvalue decomposition on the covariance matrix to obtain eigenvalues and eigenvectors, and selected three principal components (PCA Factors) to further correlation analysis. The component axes were rotated using the varimax method to better align positioning vectors and simplify the interpretation of factor loadings. We then assessed the correlations between the principal components and the expression levels of SEMA7A, SEMA4D, ADAM8, and ADAMTS10. Due to the data distribution, we chose Spearman’s rank correlation for correlation analysis. In Spearman’s rank correlation analysis, significance was determined using *p*-values with thresholds set at *p* < 0.05 for statistical significance. PCA was conducted using R Studio software by applying the “factoextra” library.

### 2.6. Gene Set Enrichment Analysis (GSEA) for SEMA7A, SEMA4D, ADAM8 and ADAMTS10 in CRC

The data used in the GSEA were derived from the “FieldEffectCrc” dataset [19]. This dataset contains CRC samples from cohort A. Based on the median expression levels of the genes SEMA7A, SEMA4D, ADAM8, and ADAMTS10, samples were categorized into “high” and “low” expression groups. Samples with expression levels equal to or above the median were classified as “high”, while the remaining samples were classified as “low”. Gene identifier mapping for the differential gene expression results was performed using the org.Hs.e.g., db database [20]. Gene sets were assigned to specific biological processes and signaling pathways based on data from the hallmark gene sets collection from MSigDB (Molecular Signatures Database) [21,22]. The analysis was conducted using R Studio software 4.4.1.

### 2.7. Statistical Analyses

Statistical analysis was conducted using R studio 2024.04.2 software (Posit PBC). The normality of the examined variables was assessed using the Shapiro–Wilk test. Data were normalized using decimal logarithm transformation. The concentrations of the investigated proteins SEMA7A, SEMA4D, ADAM8, and ADAMTS10 did not follow a normal distribution. *p*-values < 0.05 were considered statistically significant. Differences between two groups were assessed using the Mann–Whitney U-test. To investigate the association between the concentrations of the analyzed proteins with the T and N parameters of the TNM scale, as well as the disease TNM stage, we used the Kendall’s Tau rank correlation coefficient. Correlation analysis was conducted using Spearmans rank correlation test. Details regarding the PCA are discussed in Section 2.5.

## 3. Results

### 3.1. Elevated Concentration of SEMA7A, SEMA4D, ADAM8, and ADAMTS10 in CRC Tissue

**Figure 1:**
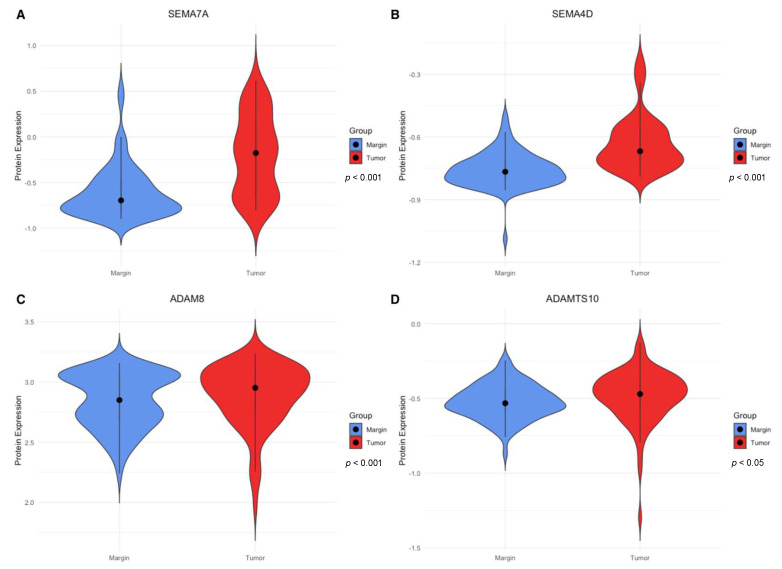
Violin plots demonstrating the concentrations of the studied proteins according to the tissue type (tumor tissue vs. surgical margin tissue). The data presented in the plots are normalized using decimal logarithmic transformation. Plot (**A**) shows differences in SEMA7A expression between the examined groups, Plot (**B**) shows differences in SEMA4D expression between the examined groups, Plot (**C**) shows differences in ADAM8 expression between the examined groups, and Plot (**D**) shows differences in ADAMTS10 expression between the examined groups. The *p*-value is the result of the U-Mann–Whitney test.

The obtained concentration of the studied proteins in CRC tissues and surgical margin tissues allowed us to investigate whether there are differences in the concentrations of the analyzed proteins between tumor and margin tissue group. We observed significantly elevated concentrations of the proteins SEMA7A (Figure 1A), (*p* < 0.001), SEMA4D (Figure 1B), (*p* < 0.001), ADAM8 (Figure 1C), (*p* < 0.001), and ADAMTS10 (Figure 1D), (*p* < 0.05) in CRC tissues compared to margin tissues.

### 3.2. Associations between Expression of SEMA7A, SEMA4D, ADAM8, ADAMTS10, Clinicopathological Parameters, and MSI Status

We subsequently investigated the potential associations between the expression levels of SEMA7A, SEMA4D, ADAM8, and ADAMTS10 proteins and the characteristics of tumor size (T), lymph node involvement (N), metastasis (M), and disease stage. Our analysis did not reveal any significant associations between the concentrations of the examined proteins and the TNM classification parameters, or the overall stage of the disease (Table 4).

We also examined whether the expression levels of the analyzed proteins might depend on the other various parameters, including tumor grading, the anatomical location of the primary tumor (classified as left-sided [L-tumors] and right-sided [R-tumors]), and microsatellite instability (MSI) status. We did not observe any significant associations between the expression of SEMA7A, SEMA4D, ADAM8, and ADAMTS10 proteins and these parameters, as detailed in Table 5.

### 3.3. Mutational Landscape in CRC Tumors Cohort and the Expression of the Examined Proteins

Further, we have determined the mutation statuses of the KRAS, NRAS, BRAF, PIK3CA, and AKT genes in the CRC tumors. The obtained gene mutation statuses enabled us to investigate whether there are differences in concentrations of the examined proteins depending on the positive status of the gene mutations. Additionally, the mutation analysis allowed us to determine the frequency of the respective mutations in the analyzed genes within the study group. Detailed information about evaluated gene mutations has been presented in Section 2 (Table 2).

The study group comprised 54 CRC tumors. KRAS gene mutations were observed in 33.33% of tumors (*n* = 18), NRAS gene mutations in 18.52% of tumors (*n* = 10), and BRAF gene mutations in 7.41% of tumors (*n* = 4), while positive mutation statuses for the PIK3CA and AKT genes were observed in 7.4% and 1.85% of tumors, respectively (*n* = 4, and *n* = 1). Among the evaluated KRAS gene mutations, the KRAS 12/13 mutation was the most frequent, constituting 61.11% (*n* = 11) of all examined KRAS mutations, whereas the KRAS 146 and KRAS 61 mutations were the least frequent, each occurring at a frequency of 11.11% (*n* = 2) among all KRAS mutations. Both NRAS gene mutations included in the analysis (NRAS 12/13 and NRAS 61) were present with equal frequency, each constituting 50% (*n* = 5) of all NRAS mutations. The studied PIK3CA gene mutations included PIK3CA 542/545 and PIK3CA 1047, with frequencies among PIK3CA mutations of 75% (*n* = 3) and 25% (*n* = 1), respectively. The distribution of the gene mutations in the study group, including detailed mutations of the respective genes, is presented in Table 6.

**Figure 2:**
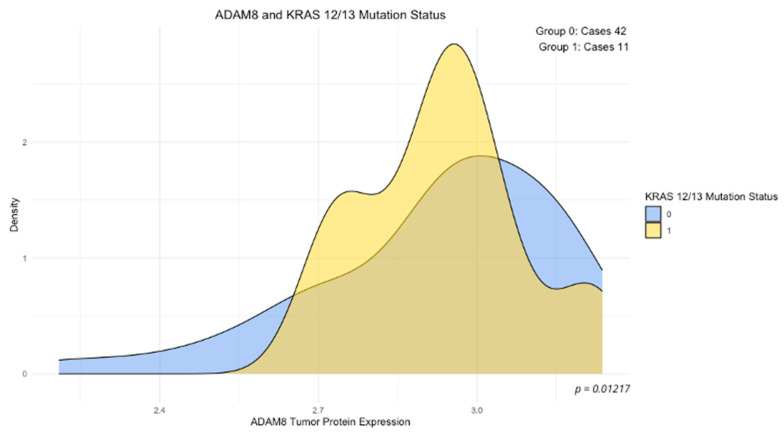
ADAM8 expression and KRAS 12/13 mutation status. Density plot: 0—KRAS 12/13 wild-type tumor, 1—KRAS 12/13 mutant tumor, *p*-value—U-Mann–Whitney test.

**Figure 3:**
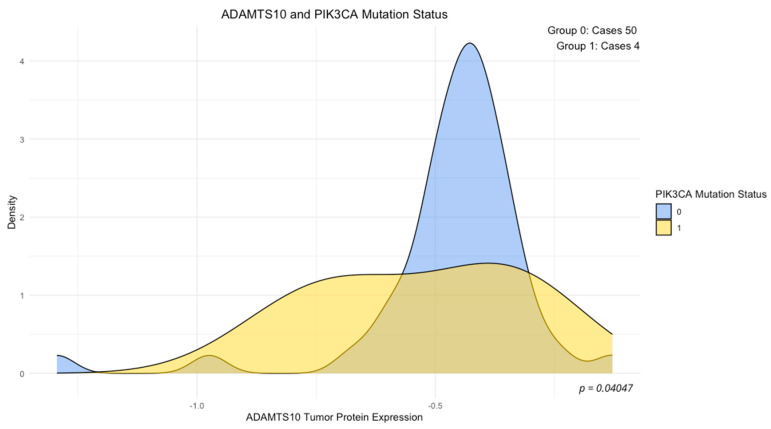
ADAMTS10 expression and PIK3CA mutation status. Density plot: 0—PIK3CA wild-type tumor, 1—PIK3CA mutant tumor, *p*-value—U-Mann–Whitney test.

**Figure 4:**
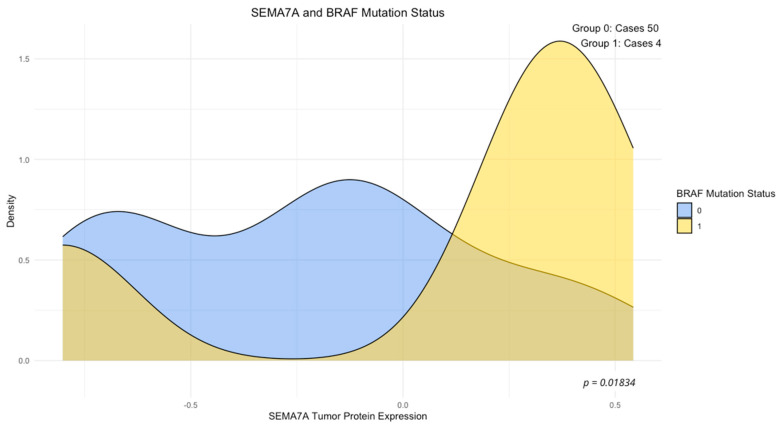
SEMA7A expression and BRAF mutation status. Density plot: 0—BRAF wild-type tumor, 1—BRAF mutant tumor, *p*-value—U-Mann–Whitney test.

The analysis of the association between concentrations of the examined proteins and mutation status of the studied genes (wild-type vs. mutant) revealed a significantly elevated expression of ADAM8 in the group of tumors with KRAS 12/13 mutations (*p* < 0.05, Figure 2), a significantly reduced expression of ADAMTS10 in the group of tumors with PIK3CA mutations (*p* < 0.05, Figure 3), and elevated concentrations of SEMA7A in the group of tumors with BRAF mutations (*p* < 0.05, Figure 4). No other significant differences in the expression levels of SEMA7A, SEMA4D, ADAM8, and ADAMTS10 were observed based on the status of the other studied mutations. In the KRAS-mutant group, ADAM8 expression appeared to be elevated in the KRAS-mutant tumor group compared to the KRAS wild-type tumor group. However, the result of the U-Mann–Whitney test did not reach statistical significance (*p* = 0.059).

### 3.4. Correlations between the Expression of SEMA7A, SEMA4D, ADAM8, and ADAMTS10

Due to the potential associations between SEMA, ADAM, and ADAMTS within the potential expression patterns or interactions between the activities of this proteins, we investigate whether there are correlations between the expression of these molecules. For SEMA7A, we observed a negative correlation with ADAM8 expression (*p* < 0.01, R = −0.313) (Supplementary Material Figure S1) and a positive correlation with ADAMTS10 (*p* < 0.05, R = 0.265) (Supplementary Material Figure S2). We also observed a positive correlation between SEMA4D and ADAMTS10 expression (*p* < 0.05, R = 0.272) (Supplementary Material Figure S3). Additionally, the expression levels of SEMA7A and SEMA4D significantly correlated with each other (*p* < 0.05, R = 0.276) (Supplementary Material Figure S4).

### 3.5. Principal Component Analysis (PCA) for Cytokine Screening Panel

**Figure 5:**
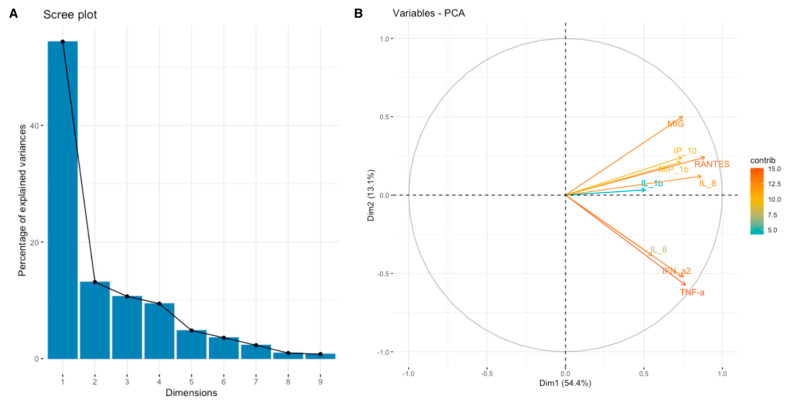
Scree plot (**A**) and biplot (**B**) for Toll-like signaling pathway set of cytokines.

**Figure 6:**
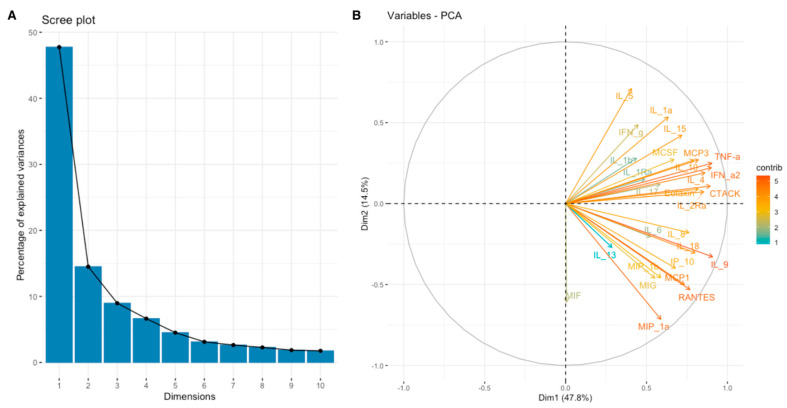
Scree plot (**A**) and biplot (**B**) for inflammatory response set of cytokines.

**Figure 7:**
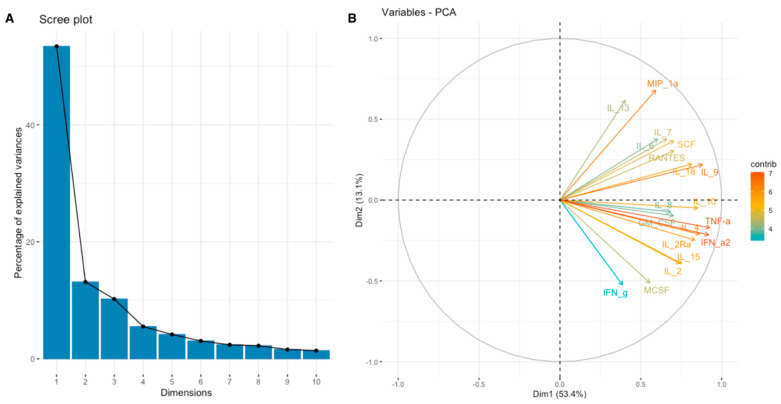
Scree plot (**A**) and biplot (**B**) for the leukocyte activation set of cytokines.

For a more comprehensive understanding of the relationship between the expression of SEMA7A, SEMA4D, ADAM8, and ADAMTS10 proteins and the expression of other cytokines, chemokines, and growth factors in the TME, we have used data obtained through the Bio-Plex Pro Human Cytokine Screening Panel to perform principal component analysis (PCA). Subsequently, cytokines, chemokines, and growth factors were assigned to the proper processes using relevant Gene Ontology (GO) terms and KEGG pathways annotations. We present examined sets of molecules from the cytokine screening panel assigned to the proper terms in Table 3 in Section 2. Further, we analyzed whether there were associations between the expression of SEMA7A, SEMA4D, ADAM8, and ADAMTS10 with the principal components (PCA Factors) obtained from PCA for the respective sets of cytokines, chemokines, and growth factors. Among the examined processes, the analyses demonstrated that SEMA4D expression was associated with the expression of cytokines, chemokines, and growth factors linked to the Toll-like signaling pathway, inflammatory response, and leukocyte activation processes. We observed a positive correlation between SEMA4D expression and Toll-like signaling pathway Factor 1 (*p* < 0.05, R = 0.3886) (Supplementary Material Figure S5), inflammatory response Factor 1 (*p* < 0.05, R = 0.399) (Supplementary Material Figure S6), and leukocyte activation Factor 3 (*p* < 0.05, R = 0.4803) (Supplementary Material Figure S7). The eigenvalue and percentages of variance for the three PCA Factors of Toll-like signaling pathway, inflammatory response, and leukocyte activation cytokine sets are presented in Table 7. The coordinates for the variables for each set are presented in Table S1 in Supplementary Material. Scree plots and biplots for examined terms are presented in Figure 5, Figure 6 and Figure 7.

**Figure 8:**
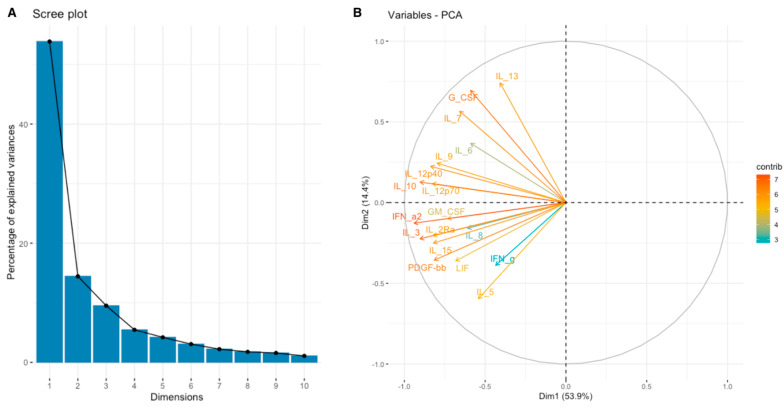
Scree plot (**A**) and biplot (**B**) for JAK-STAT signaling pathway set of cytokines.

**Figure 9:**
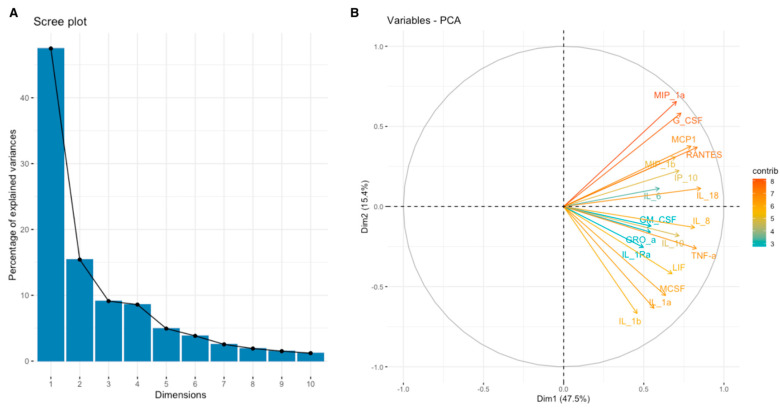
Scree plot (**A**) and biplot (**B**) for Interleukin-10 signaling set of cytokines.

**Figure 10:**
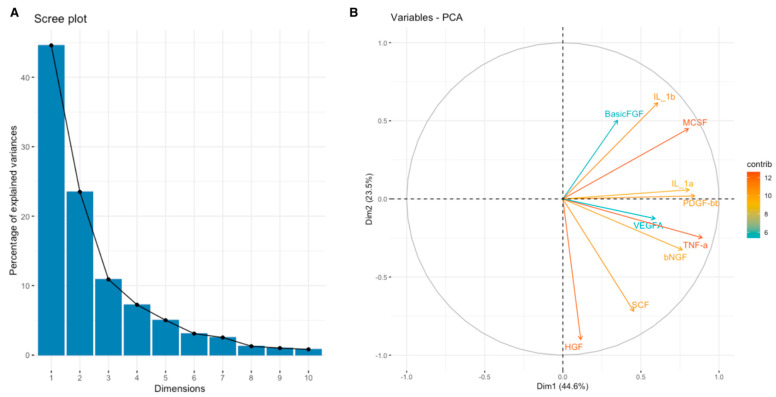
Scree plot (**A**) and biplot (**B**) for MAPK signaling pathway set of cytokines.

The analysis of SEMA7A expression revealed a significant positive correlation between SEMA7A expression and PCA Factor 1 for the JAK-STAT signaling pathway (*p* < 0.05, R = 0.39) (Supplementary Material Figure S8), PCA Factor 3 for IL-10 signaling (*p* < 0.05, R = 0.39) (Supplementary Material Figure S9), and PCA Factor 3 for the MAPK signaling pathway (*p* < 0.05, R = 0.48) (Supplementary Material Figure S10). The eigenvalue and percentages of variance for the three PCA Factors of the JAK-STAT signaling pathway, Interleukin-10 signaling, and MAPK signaling pathway cytokine sets are presented in Table 8. Coordinates for the variables for examined sets are presented in Table S2 in the Supplementary Material. Scree plots and biplots for appropriate processes are presented in Figure 8, Figure 9 and Figure 10.

We did not observe any significant associations between the expression of ADAM8, ADAMTS10, and the processes included in the PCA.

### 3.6. Gene Set Enrichment Analysis (GSEA) for High vs. Low SEMA7A, SEMA4D, ADAM8, ADAMTS10 Gene Expression in CRC Tumors

**Figure 11:**
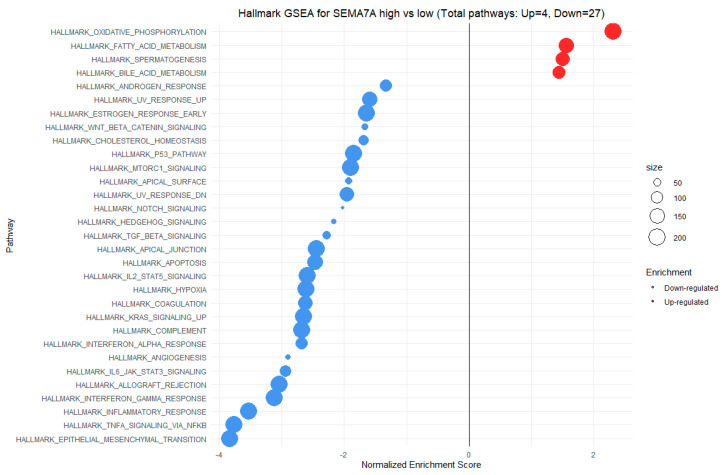
GSEA for high vs low SEMA7A expression. The X-axis represents the NES (Normalized Enrichment Score), while the Y-axis displays various biological processes and signaling pathways from the hallmark gene sets collection from MSigDB (Molecular Signatures Database). The size of the points illustrates the number of genes with dysregulated expression for proper hallmark gene sets in the high vs. low SEMA7A expression groups, as depicted in the accompanying legend at the right side.

Gene Set Enrichment Analysis (GSEA) indicates that hallmark gene sets enriched in the group with high SEMA7A expression (with positive NES score) were mainly associated with metabolic processes. These processes include oxidative phosphorylation, fatty acid metabolism, and bile acid metabolism. The hallmark gene sets with negative NES scores for SEMA7A expression are primarily associated with inflammation, cytokine signaling, and angiogenesis. These processes include MTORC1 signaling, TGF-beta signaling, IL-2/STAT5 signaling, interferon-alpha response, interferon-gamma response, inflammatory response, and angiogenesis, among others. Additionally, in the low SEMA7A expression group, enriched hallmark gene sets include epithelial–mesenchymal transition, hedgehog signaling, and KRAS signaling upregulation (Figure 11).

**Figure 12:**
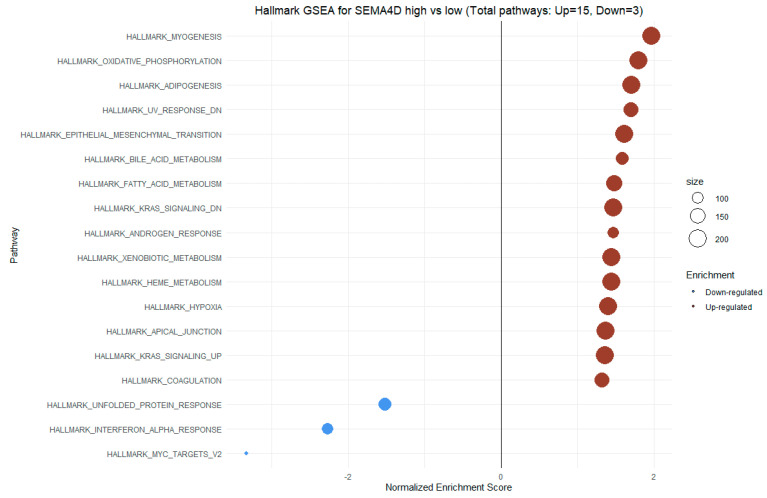
GSEA for high vs low SEMA4D expression. The X-axis represents the NES (Normalized Enrichment Score), while the Y-axis displays various biological processes and signaling pathways from the hallmark gene sets collection from MSigDB (Molecular Signatures Database). The size of the points illustrates the number of genes with dysregulated expression for proper hallmark gene sets in the high vs. low SEMA4D expression groups, as depicted in the accompanying legend at the right side.

For high SEMA4D expression, we observed enrichment in hallmark gene sets such as myogenesis, oxidative phosphorylation, adipogenesis, epithelial–mesenchymal transition, fatty acid metabolism, and KRAS signaling regulation, among others. Negative NES scores for SEMA4D expression were observed for hallmark gene sets like unfolded protein response, interferon-alpha response, and MYC-targets V2 (Figure 12).

**Figure 13:**
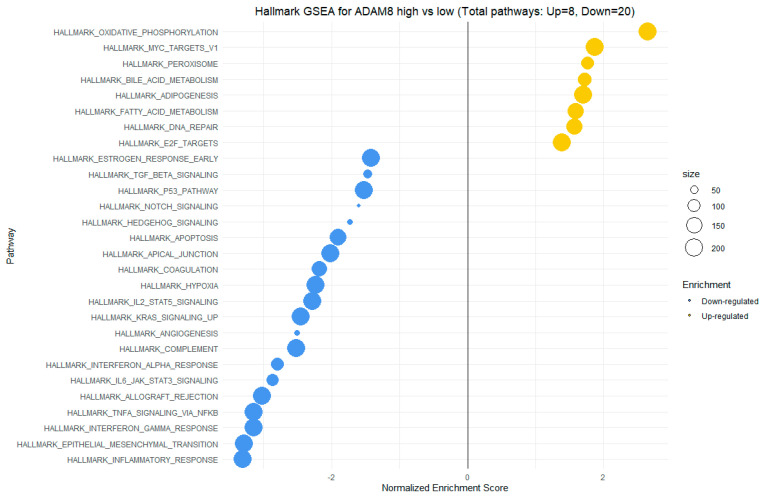
GSEA for high vs low ADAM8 expression. The X-axis represents the NES (Normalized Enrichment Score), while the Y-axis displays various biological processes and signaling pathways from the hallmark gene sets collection from MSigDB (Molecular Signatures Database). The size of the points illustrates the number of genes with dysregulated expression for proper hallmark gene sets in the high vs low ADAM8 expression groups, as depicted in the accompanying legend at the right side.

GSEA for ADAM8 high vs low expression revealed enrichment in hallmark gene sets such as oxidative phosphorylation, MYC-targets V1, peroxisome, fatty acid metabolism, adipogenesis, and others, in high ADAM8 expression. Hallmark gene sets with negative NES scores for ADAM8 expression included NOTCH signaling, apoptosis, IL-2/STAT5 signaling, KRAS signaling regulation, interferon-alpha response, epithelial–mesenchymal transition, and inflammatory response, among others (Figure 13).

**Figure 14:**
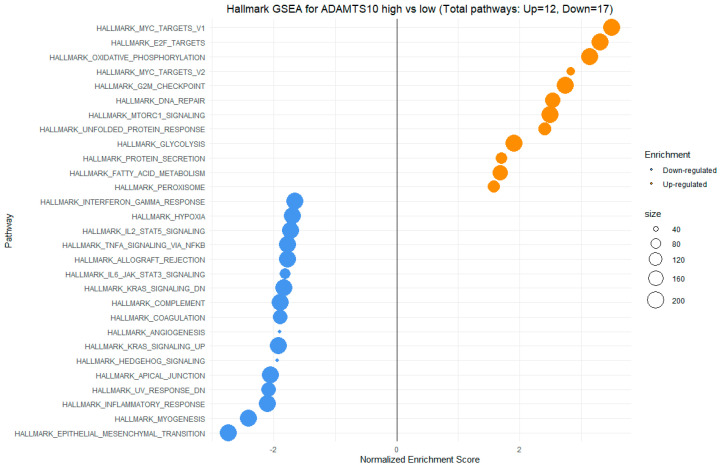
GSEA for high vs low ADAMTS10 expression. The X-axis represents the NES (Normalized Enrichment Score), while the Y-axis displays various biological processes and signaling pathways from the hallmark gene sets collection from MSigDB (Molecular Signatures Database). The size of the points illustrates the number of genes with dysregulated expression for proper hallmark gene sets in the high vs low ADAMTS10 expression groups, as depicted in the accompanying legend at the right side.

GSEA also indicates enrichment in hallmark gene sets such as MYC-targets V1, oxidative phosphorylation, MYC-targets V2, MTORC1 signaling, fatty acid metabolism, G2M checkpoint, and others in the group with high ADAMTS10 expression. Negative NES scores obtained from GSEA for ADAMTS10 gene expression were observed for interferon-gamma signaling, angiogenesis, inflammatory response, epithelial–mesenchymal transition, IL-2/STAT5 signaling, and others (Figure 14).

## 4. Discussion

According to Globocan data, by 2040, the number of CRC cases is predicted to increase to 3.2 million new cases [23]. Currently, despite a noticeable decrease in the age-standardized mortality rate in highly developed countries, CRC remains the second-leading cause of cancer-related deaths [24]. Despite the significant effects of screening and advancements in available therapeutic options, at diagnosis, 25% of people present with stage IV disease, and nearly 60% of patients with stage II-III CRC will develop post-operative recurrence [25]. Further progress in the treatment of advanced CRC may be achieved through the continued development of precision oncology, which has seen intensive growth over the past decades. The proposed classifications of CRC tumors, such as The Consensus Molecular Subtype (CMS) [26] or the IMF (Intrinsic, Microsatellite, Fibrosis) system proposed in 2022 [27], may encounter several difficulties before they transition into routine clinical use and before it becomes part of CRC treatment guidelines. In terms of matching appropriate treatment methods and better understanding the molecular characteristics of CRC tumors based on the constellation of commonly occurring gene mutations, there is still so much to explore.

According to the ESMO Clinical Practice Guidelines, testing for mismatch repair (MMR) genes status and mutations in KRAS, NRAS (exons 2, 3, and 4), and BRAF is recommended for all patients at the time of metastatic CRC (mCRC) diagnosis [28]. Mutations in the KRAS, NRAS, BRAF, and PIK3CA genes significantly impact the effectiveness of applied treatment methods and disease progression [2,29]. The KRAS gene is mutated in about 30–50% of CRC cases, making it the most frequently mutated oncogene in this cancer [30,31]. NRAS gene mutations are less common, occurring in approximately 3–10% of CRC cases [32,33]. The most frequently observed mutations in the KRAS gene are in exon 2, at codon 12, followed by codon 13. Mutations in exons 3 and 4 are less frequently encountered [34,35]. According to studies by Zeng et al., among 408 CRC patients, 33.1% (135) had KRAS mutations in exon 2, while NRAS mutations in exons 2 and 3 were found in 2.4% of patients (*n* = 10) [36]. Benmokhtar et al. also observed similar frequencies of mutations in the respective exons, noting over 90% of KRAS mutations in exon 2 (68.9% in codon 12, 24.4% in codon 13), and an increased frequency of NRAS mutations compared to Zeng et al., with 8.8% and mutation rates in exons 2, 3, and 4 at 57.1%, 28.6%, and 14.3%, respectively [37]. KRAS and NRAS are variants of the RAS genes, which are significant in CRC pathology, not only due to their high frequency in this malignancy but also because of the processes they regulate. RAS genes encode small GTPase proteins involved in several signaling pathways that ultimately regulate cell growth, motility, angiogenesis, and survival. Moreover, oncogenic KRAS induces several inflammatory cytokines, chemokines, and plays a critical role in shaping the immune TME through KRAS-downstream pathways [38,39]. In our cohort of 54 patients randomly selected for mutational landscape analysis, KRAS mutations in exon 2 (corresponding to KRAS 12/13 mutations), similar to previously mentioned studies, were the most frequently observed among KRAS gene mutations, constituting 61.11% of KRAS mutations (Table 6). For mutations in the other studied exons, 3 and 4, their frequencies were higher in our study than in Zeng et al. In our study, the frequency of mutations in these exons was 9% each, while in Zeng et al., they were 2.7% and 3.7%, respectively [36]. Similarly, we observed a higher frequency of NRAS mutations compared to Zeng et al. (about 2.5% of mutations in the entire study group) and Benmokhtar et al. (about 8.8% of NRAS mutations in the entire study group), as in our study, it was approximately 18%, with equal frequencies of NRAS mutations in exons 2 and 3 [36,37]. These observed differences may be due to the distinction in the age of patients qualified for the study, disparity in the proportion of men and women in the study group, differences in the distribution of patients with advanced tumor stage, and differences in ethnicity and race. The only association between the levels of expression of the studied proteins with the examined KRAS mutations was significantly elevated ADAM8 protein concentration in the group of KRAS 12/13 tumors. KRAS mutation through downstream pathways such as MAPK/ERK can influence the regulation of gene expression involved in cell motility, invasiveness, and survival behaviors. ADAM8, being an extracellular matrix protease, can be regulated by these pathways, leading to its elevated expression in the KRAS 12/13-mutated tumor group [40]. Tumors with KRAS 12/13 mutations may exhibit a more aggressive tumor phenotype potentially due to the significant association of ADAM8 with the formation of distant metastases and the invasion of the tumor environment [41,42].

Mutations in the BRAF gene, which encode a serine/threonine protein kinase, are detected in 7–10% of CRC patients [43,44]. BRAF mutations frequently co-occur with MSI-high status tumors and are associated with poorer prognosis [45,46]. The most commonly studied BRAF gene mutation is V600E, which is the most frequently occurring BRAF gene mutation. Consistent data indicate that the BRAF V600E mutation is associated with poor outcomes after relapse [47,48]. In our study, we examined BRAF gene mutations at the exon 15. Among the studied mutations, besides V600E, we also examined V600E2, V600D, and V600K mutations (Table 2). BRAF gene mutation in our study occurred with a frequency of about 7%, which reflects the findings of other studies. However, due to examining mutations jointly for exon 15, we cannot determine the frequency of mutations in the respective codons for the BRAF gene in our study. Among the studied proteins in our study, we observed significantly elevated levels of SEMA7A protein in BRAF-mutant tumors compared to the BRAF wild-type tumor group. It appears that the BRAF gene mutation, particularly BRAF V600E, which leads to the constitutive activation of the MAPK/ERK pathway, may influence the expression of pro-inflammatory cytokines and modulate the functions of macrophages and T cells, leading to an increased expression of SEMA family proteins [49,50]. The correlation between the presence of the BRAF V600E mutation and SEMA family proteins was observed by Loria et al. They demonstrated that SEMA6A expression is significantly elevated in human melanoma cells and that SEMA6A silencing in BRAF-mutant melanoma cells led to impaired cell growth, motility, and invasiveness [51]. The observed correlation in our study is a preliminary finding regarding the relationship between the BRAF gene mutation and elevated SEMA7A protein expression, which, in addition to verification in a larger study group, requires further evaluation in terms of regulatory processes in the CRC microenvironment that are influenced by increased SEMA7A expression in BRAF-mutant CRC.

The PIK3CA and AKT1 genes are involved in the PI3K/AKT signaling pathway, which influences multiple pathways related to carcinogenesis and cancer progression. PIK3CA gene mutations occur in 10–30% of CRC tumors, whereas AKT1 gene mutations are rare, with an incidence of around 1% [52,53,54]. In our group, the PIK3CA mutation studied in exons 9 and 20 occurred with a frequency of about 7%, which is lower than observed in other studies. The only association we observed in our study between PIK3CA mutation status and the levels of studied proteins was the decreased ADAMTS10 protein concentration in the PIK3CA-mutated tumor group (Figure 3). Considering the impact of constitutive activation of the PI3K/AKT/mTOR pathway on various directions of the regulation of various transcription factor, the PIK3CA mutation may lead to decreased ADAMTS10 expression. On the other hand, considering the impact of PI3K/AKT/mTOR pathway activation on interactions in the CRC TME, affecting the increased activity of extracellular matrix proteases, this observation requires verification in studies involving a larger group of patients with PIK3CA-mutant tumors [29]. In the case of this group of tumors, due to the association between SEMA4D expression and the PI3K/AKT pathway, a correlation with SEMA4D was expected. The role of SEMA4D in the creation of the CRC TME properties heavily depends on its influence on the PI3K/AKT signaling pathway [55]. However, such an association was not observed.

MSI occurs in approximately 15% of sporadic CRC cases and results from deficient DNA MMR genes due to either germline mutations in MMR genes (MLH1, MSH2, MSH6, PMS2) or the epigenetic inactivation of MLH1 [56,57]. Colorectal tumors with high MSI (MSI-high) are especially sensitive to immunotherapy [58]. In our study group, the proportion of MSI-high tumors was nearly 18% (Table 1), which is similar to the frequency described in the other publications considering MSI status in sporadic CRC. In our study, we did not observe significant differences in the levels of SEMA7A, SEMA4D, ADAM8, and ADAMTS10 between the MSI-high and MSI-low groups. In both groups, the levels of these proteins were significantly elevated compared to their expression in margin tissue. This may indicate that the elevated expression of both the studied SEMAs, ADAM8, and ADAMTS10 is induced by gene mutations and molecular events occurred during the progression of both MSI-low and MSI-high CRC tumors. Alternatively, mutations in MMR genes for MSI-high may directly cause an increased expression of the studied proteins or, through strong infiltration of immune cells and increased activity of relevant signaling pathways, lead to an elevation similar to that in MSI-low tumors, where microsatellite instability is lower. Other mechanisms, such as epigenetic changes or a constellation of mutations in genes other than MMR, could lead to the upregulation of the studied proteins. Nonetheless, an important question for future studies is whether the proteins SEMA7A, SEMA4D, ADAM8, and ADAMTS10 perform the same functions in the MSI-high and MSI-low CRC groups.

SEMA7A, similar to other members of the SEMA family, was initially considered an axon-guidance molecule [59]. SEMA7A is composed of a seven-bladed β-propeller semaphorin N-terminus domain, an immunoglobulin-like domain, a plexin, semaphorin, and integrin domain (PSI), and a characteristic GPI anchor in its C-terminus. Its specific receptor is PlexinC1 [60]. However, further research has shown its significance in immune response modulation, affecting dendritic cells, monocytes, eosinophils, and T cells; SEMA7A can effectively stimulate monocytes or macrophages to produce the following pro-inflammatory cytokines: IL1β, TNF-α, IL6, and IL8 [61,62,63]. Delorme et al. also highlight SEMA7A’s role in the differentiation and chemotaxis of the aforementioned immune cells [64]. Due to its significance in managing immune responses, SEMA7A is involved in the pathogenesis of various diseases: rheumatoid arthritis [65], multiple sclerosis [66], pulmonary fibrosis [67], liver fibrogenesis [68], and multiple types of cancer, including breast [69], pancreatic [70], CRC [71], gastric [72], melanoma, and oral squamous cell carcinoma [61].

Semaphorin 4D (also called CD100) is a member of the SEMA4 transmembrane glycoprotein family. The protein contains the following parts: NH2 signal sequence, a SEMA domain, a polylysine stretch, an Ig domain, a transmembrane domain, and an atypical cytoplasmic tail. Regarding receptors, scientists are aware of two members of the plexin family: PlexinB1 and PlexinB2, and CD72. The presence of this protein has been observed in numerous tissues, including the brain, kidney, heart, bones, thymus, and colon. Moreover, a significant number of tumors express elevated levels of this protein, including breast, ovarian, cervical, and CRCs [73,74,75]. Its activation through the SEMA4D/PlexinB1 axis results in the increased proliferation of acute myeloid leukemia cells, the increased metastasis and growth of cancer cells in osteosarcoma, bladder cancer, pancreatic cancer, prostate cancer, and can even reduce the effectiveness of nivolumab in melanoma therapy [55,76,77]. Increased levels of SEMA4D are known to be present in CRC, leading to elevated cell propagation, aggressiveness, and metastatic potential. Silencing SEMA4D results in the decreased migration and overall aggressiveness of CRC, highlighting its possible therapeutic role in the future [75,78,79]. The exact role of SEMAs in modulating the immune system in CRC is unknown. SEMA4D’s role is especially ambiguous. In a variety of diseases (Kawasaki disease, multiple sclerosis, and rheumatoid arthritis), the SEMA4D/CD72 axis leads to increased levels of cytokines and results in worse prognosis. However, there are reports of SEMA4D’s inhibitory role in cytokine-driven immune responses, where cytokine levels (TNFα, IL-6, IL-1β) were downregulated [80,81,82].

ADAM8 is considered to be an important factor in the development of cancer. It has been revealed that ADAM8 activates the integrin pathway through β1-integrin, increases angiogenesis through the shedding of many angiogenesis-related proteins, and promotes invasiveness by activating the MAPK signaling pathway [83]. ADAMs are connected to many different diseases. For example, ADAM10 was shown to be involved in neurodegenerative diseases, autoimmune diseases, atherosclerosis, and cancer [84]. However, other ADAMs are also mentioned in connection with other diseases such as rheumatoid arthritis, diabetes, multiple sclerosis, asthma, and Alzheimers disease [8,85]. A number of different ADAMs were shown to be involved in the migration, invasion, and proliferation in different cancer types such as breast cancer, gastrointestinal cancer, or brain cancer [3,85,86]. ADAM17 was reported to be involved in many different processes within cells, through mechanisms such as the phosphorylation of EGFR, the cleavage of ACE2, and the inactivation of AMPK or shedding of receptors for TNF-α [87,88]. Similarly, ADAM10 was shown to be able to cleave a number of substrates such as PD-L1 or EGFR/HER ligands [89]. It is also worth adding that the involvement of ADAM10 in the Notch signaling pathway [3]. ADAM8 and ADAMTS10 have not yet been described in the context of KRAS, NRAS, BRAF, PIK3CA, or AKT mutations and pathways; however, other ADAMSTs have been. For example, the antitumor effects of ADAMTSs can be mediated through impaired ERK phosphorylation [9]. In THP-1 cells, ADAMTS5 was shown to be upregulated by IL-17A through the ERK and JNK pathways. Furthermore, ADAMTS-1 was reported to inhibit angiogenesis in lung cancer cells by regulating the PI3K/Akt-eNOS/VEGF axis [90]. In Triple-negative breast cancer, ADAM8 was able to induce miR-720 by activating a β1-integrin to the ERK signaling pathway [91]. In another study, Liu et al. reported the ability of ADAM8 to activate HB-EGF/EGFR signaling, thereby increasing the production of CCL2 in glioblastoma cells treated with TMZ [92]. Furthermore, in a similar environment, ADAM8 was shown to cause chemoresistance by increasing pAkt/PI3K and pERK1/2 [93]. ADAMTS10 mutations cause Weill–Marchesani syndrome (WMS), suggesting its role in fibrillin microfibril biology, as some fibrillin-1 mutations also cause WMS [94]. ADAMTSs have been connected to many diseases apart from WMS. ADAMTS13 is associated with thrombotic thrombocytopenic purpura, ADAMTS5 with osteoarthritis, and ADAMTS7 is associated with atherosclerosis and coronary artery disease [95]. Other ADAMTSs have also been shown to be possibly associated with cardiovascular disease [96]. ADAMTS7 is considered to be involved in inflammatory diseases [97]. It is worth mentioning the possible involvement of ADAMTSs in central nervous system homeostasis, supposedly through degradation and interaction with components of the ECM [98]. These studies show the importance and versatility of ADAMTS proteins in various biological processes. Their expression has been connected with carcinogenesis, particularly events such as cell adhesion, migration, proliferation, and angiogenesis [9]. Sun et al. proposed three different mechanisms by which ADAMTSs can impact angiogenesis. The first relies on the cleavage of the large thrombospondin (TSP)1 and TSP2, resulting in the release of the anti-angiogenic 3TSR region from the ECM. The next involves the sequestration of VEGF, subsequently affecting VEGFR2 receptor phosphorylation. The last is based on the cleavage of the von Willebrand factor (vWF), resulting in optimal coagulation and angiogenesis [99]. Another mechanism worth mentioning is the ability of ADAMTS to cleave proteoglycans. Such processes usually result in tumor suppression [100]. A number of ADAMTS proteins have been shown to have altered expression in CRC tissue, suggesting their role in this type of tumor [101]. Another study by Li et al. showed that ADAMTS12 can alter the Wnt/β-catenin pathway, which is known for its importance in CRC carcinogenesis [102]. ADAMTS10 was reported to alter the JAK/STAT/c-MYC pathway and reprogram macrophages in gastric cancer, possibly inhibiting cancer progression, and was shown to be a plausible prognostic biomarker [103]. In clear cell renal carcinoma, ADAMTS10 was upregulated, and its inhibition resulted in the impaired ability of cells to proliferate, invade, and migrate. Furthermore, it was connected to the NF-κB signaling pathway [104]. Both ADAM and ADAMTS enzymes are known for their contribution to inflammation. It has been shown that they can alter the expression of a number of cytokines. ADAMTS1 was associated with TGF-β expression, ADAMTS-13 was negatively correlated with C-reactive protein (CRP), IL-6, TNFα, and IL-1β, and ADAMTS2 was regulated by IL-6 [105,106,107]. A number of studies have shown that cytokines can significantly alter the expression of ADAM8 [108]. Moreover, ADAM8 was reported to be associated with the increased release of pro-inflammatory cytokines such as TNF-α and IL-6 [109].

In our study, we observed significant positive correlations between the expression of SEMA7A and ADAMTS10 (Figure 6), SEMA4D and ADAMTS10 (Figure 8), and between the expression of SEMA7A and SEMA4D (Figure 7), as well as a significant negative correlation between the expression of SEMA7A and ADAM8 (Figure 5). Associations between the expression of SEMA proteins and proteins from the ADAM and ADAMTS families have been poorly studied to date. Esselens et al. demonstrated that the overexpression of ADAMTS1 led to the release of SEMA3C from the extracellular matrix in breast cancer cell models and that this activation of SEMA3C was particularly associated with an increased migration of breast cancer cells, contributing to the promotion of metastasis formation [110]. A similar effect on the formation of soluble forms of SEMA family proteins was demonstrated by Motani et al. for SEMA4D by ADAM17 in studies on the role of these proteins during the activation of the stimulator of interferon genes (STING). They showed that STING activated ADAM17, which caused the formation of the soluble form of SEMA4D, which could perform pro-inflammatory functions in macrophage cultures [111]. The expression of SEMA and ADAM can be regulated by the activity of similar pathways, as demonstrated in the case of activin A in hepatocytes for SEMA7A and ADAM12 by Haridoss et al. [112]. Correlations between the expression of SEMA5A and ADAM17 were also observed by Du et al. in systemic lupus erythematosus (SLE), where their study results suggested the involvement of ADAM17 in the secretion process of SEMA5A [113]. The dependencies we observed between the expression of SEMA7A, SEMA4D, ADAM8, and ADAMTS10 seem to indicate the regulation of these proteins’ expression by similar transcription factors and signaling pathways, which may be a response to cellular stress, hypoxia, or a pro-inflammatory response in the tumor microenvironment. The exact molecular interactions between the respective domains of SEMA and ADAM proteins, as well as understanding the correlations between the functions these proteins perform in the CRC progression, remain open questions. The observed correlations of SEMA4D expression with factors involved in the immune and inflammatory response suggest that SEMA4D may play an important role as a mediator of these processes. In our study, we observed a positive correlation between SEMA4D expression and the principal components for groups of cytokines, chemokines, and growth factors associated with the Toll-like signaling pathway, inflammatory response, and leukocyte activation. In the case of SEMA7A, the only dependencies observed in the PCA included positive correlations of SEMA7A expression with processes in the JAK-STAT signaling pathway, IL-10 signaling, and MAPK signaling. However, the observed results are not fully consistent with the GSEA results for SEMA4D and SEMA7A. The GSEA results, where we compared the enrichment of signaling pathways and biological processes depending on the high and low expression groups of SEMA7A, seem to indicate an increased metabolic activity in cells with high SEMA7A expression through the enrichment of metabolic pathways such as fatty acid metabolism, oxidative phosphorylation, and the regulation of immune responses and inflammatory processes through the enrichment of hallmark gene sets such as TNF-α signaling, interferon gamma, and inflammatory response in the low SEMA7A expression group. Similar conclusions can be drawn based on the GSEA results for SEMA4D, where high SEMA4D expression was associated with the enrichment of hallmark gene sets associated with various metabolic processes such as oxidative phosphorylation, adipogensis, epithelial–mesenchymal transition, and fatty acid metabolism, while low SEMA4D expression was associated with processes such as interferon-alpha response and MYC-targets. The GSEA for ADAM8 revealed that the most upregulated pathways for the high expression of this protein were those related to metabolism and cell proliferation, such as oxidative phosphorylation, adipogenesis, and MYC targets. Conversely, pathways most enriched in the low ADAM8 expression group, such as inflammatory response and interferon gamma response, suggest a potential role of ADAM8 in the suppression of the immune response in CRC TME. However, these findings were not supported by the cytokine screening panel and ADAM8 expression analysis. The GSEA results for ADAMTS10, regarding the processes and pathways that were upregulated and downregulated, appear to be similar to those for ADAM8. Interestingly, both reduced expressions of ADAM8 and ADAMTS10 in GSEA were associated with the enrichment of the epithelial–mesenchymal transition process, indicating an inhibitory effect on the migration and invasiveness of CRC cells.

**Figure 15:**
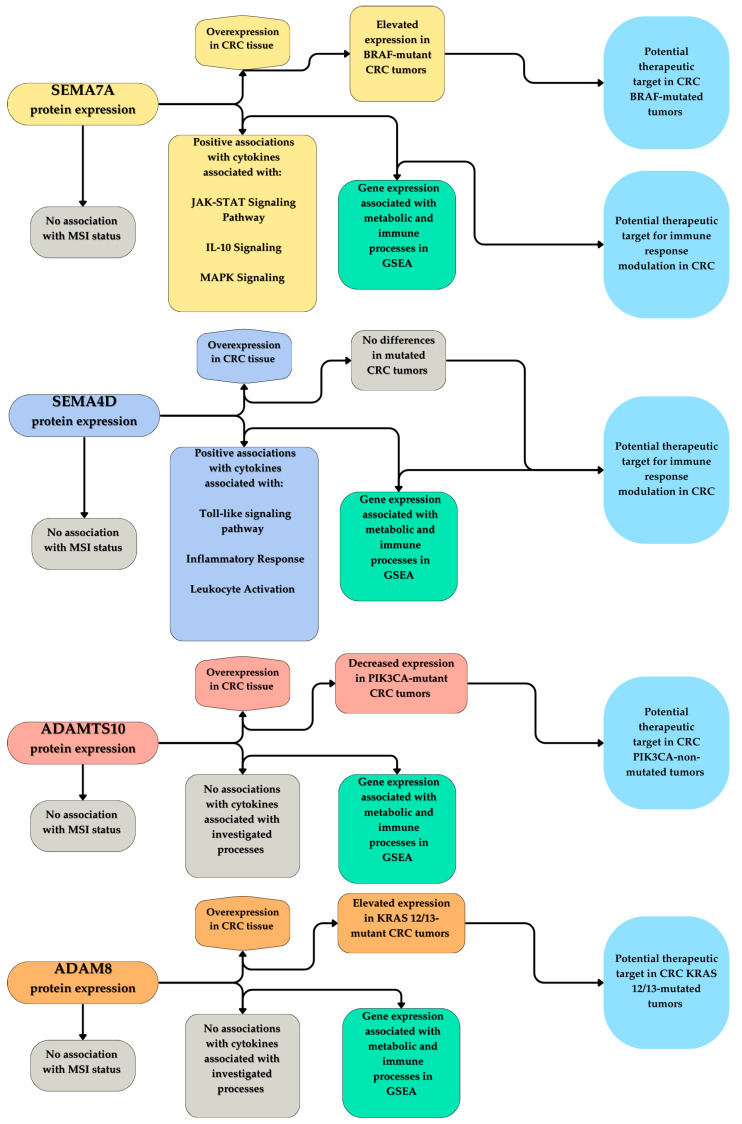
Summary of the observed results in the study for the SEMA7A, SEMA4D, ADAM8, and ADAMTS10, along with the potential clinical significance of these findings, warranting further verification in subsequent studies. CRC—colorectal cancer; MSI—microsatellite instability; GSEA—Gene Set Enrichment Analysis.

Considering the impact of researched molecules on CRC, it is possible to consider them as therapeutic targets. Their impact on many different processes within cells, and their high expression in cancer tissues in comparison to normal tissue, makes them worth exploring in terms of possible inhibition in clinical situations. It has been shown that in murine models, the silencing of SEMA7A resulted in the impaired angiogenesis, migration, proliferation, and invasiveness of breast cancer [114,115,116]. Similarly, in oral tongue squamous cell carcinoma, EMT was reported to be influenced by SEMA7A and was inhibited in an in vivo model, increasing survival [117]. Kinehara et al. observed the impact of SEMA7A on EGFR-TKI resistance [118]. Moreover, head and neck squamous cell carcinoma progression was impaired in SEMA7A knockdown cells [119]. The influence of SEMA7A on CRC has not been yet researched in terms of in vivo inhibition. SEMA4D, on the other hand, was examined in regard to CRC. Ding et al. observed the impact of in vivo silencing of SEMA4D on angiogenesis [79]. It is worth mentioning the Phase I clinical trial of Pepinemab, which is an anti-SEMA4D antibody, combined with Ipilimumab or Nivolumab in treatment of patients with resectable CRC [120]. Furthermore, in vivo studies on other types of cancer, such as acute myeloid leukemia [55], bladder cancer [121], and breast cancer [122] have shown that the inhibition of SEMA4D has therapeutic potential. Mineva et al. presented research, where two anti-ADAM8 antibodies ADP2 and ADP13 reduced aggressiveness and improved survival in mice [123]. Similarly, ADAM8 deficient glioblastoma cells had impaired angiogenesis in in vivo models [124]. Lastly, ADAM8 knockdown renal clear cell carcinoma inhibited tumor formation and increased the survival of mice [125]. ADAMTS10 is least described protein out of the four researched in this paper; therefore, in vivo studies are limited to research published by Zhou et al. showing that the increased expression of ADAMTS10 results in inhibition of gastric cancer tumor growth [103]. Considering our data and studies mentioned above, researched molecules can be considered as promising therapeutic targets for the treatment of CRC. Considering the increased expression of ADAM8 in KRAS 12/13-mutated tumors, SEMA7A in BRAF-mutated tumors, and the decreased expression of ADAMTS10 in PIK3CA-mutated tumors, it is possible that these groups of patients would be more susceptible to therapy based on ADAM8, SEMA7A, and ADAMTS10 inhibition. Therefore, it would be preferable to determine not only mutational status but also the expression of respective proteins, before inducing therapy. A summary of the study’s observed results for validation in future studies, along with the potential clinical significance to be verified in clinical trials, is presented in Figure 15.

### Study Limitations

The issues concerning the directionality of the observed dependencies between the expression of SEMA7A and SEMA4D with immune processes and the lack of observed dependencies between the expression of ADAM8 and ADAMTS10 with processes analyzed in PCA, as well as comparing the obtained results with the GSEA results for the expression of SEMA7A, SEMA4D, ADAM8, and ADAMTS10, have certain limitations. These limitations include the fact that, when analyzing the relevant processes in PCA, we considered a limited number of cytokines, chemokines, and growth factors associated with the respective processes, whereas their actual number is much larger. The large scale of the GSEA speaks for the increased accuracy of the obtained results. Due to these and other limitations of the applied research methods, we treat our results as preliminary findings. Nevertheless, both the results from our experimental studies and statistical tests, as well as the GSEA results, indicate the association between the SEMA7A, SEMA4D, ADAM8, and ADAMTS10 protein expression and the mentioned immune processes in CRC TME.

A significant limitation of the presented study is the number of tumors exhibiting a positive status of the investigated mutations. The frequency of mutations such as BRAF, PIK3CA, and AKT, which do not commonly occur in CRC patient groups, resulted in a limited number of individual tumors with positive mutation statuses in the presented cohort of 54 tumors. Although the frequency of these mutations in our study largely aligned with frequencies reported in other studies, the small number of positive cases limits the generalizability of the findings. Therefore, the observed associations between the expressions of the SEMA7A, SEMA4D, ADAM8, and ADAMTS10 in tumor groups with KRAS, NRAS, BRAF, PIK3CA, and AKT mutations require validation in larger study cohorts.

In this article, the relationships between the expression of the investigated proteins and the analyzed biological and immunological processes, represented by groups of cytokines, chemokines, and growth factors, were analyzed using PCA. Additionally, correlations among the studied molecules were assessed. However, these analyses do not allow us to precisely determine the nature of the observed associations. To further elucidate the potential regulatory interactions between the studied molecules and the expression of the examined cytokines, appropriate experiments using cell cultures or animal models, which were not conducted in this study, are necessary. To accurately assess the impact of the altered expression of SEMA7A, SEMA4D, ADAM8, and ADAMTS10 on the immunological properties of the TME, as well as the function of the studied proteins in patient groups with KRAS, BRAF, and PIK3CA mutations, further research is required. Such studies are essential for precisely evaluating the potential of targeting the expression of the investigated molecules in immunotherapy and the other targeted therapy methods.

## 5. Conclusions

The expression of SEMA7A and SEMA4D was associated with immunological and metabolic processes occurring within the tumor microenvironment. SEMA7A expression was linked to altered cytokine expression associated with the JAK-STAT signaling pathway, IL-10 signaling, and MAPK signaling, as well as genes involved in inflammatory response and TNFα signaling via NF-κB, among others. SEMA4D expression was associated with altered cytokine expression related to the Toll-like receptor signaling pathway, inflammatory response, leukocyte activation, and genes involved in the interferon-alpha response, hypoxia, and unfolded protein response, among others. The precise immunological and metabolic functions of SEMA7A, SEMA4D, as well as ADAM8 and ADAMTS10 in CRC, require further investigation.

The widespread expression observed in our study of the SEMA7A, SEMA4D, ADAM8, and ADAMTS10, depending on the mutational landscape of the KRAS, NRAS, BRAF, PIK3CA, and AKT genes, and the MSI status, along with the significant immune processes within CRC TME, in which these proteins appear to be involved, indicates that SEMA7A, SEMA4D, ADAM8, and ADAMTS10 may be considered as promising subjects for further analysis in cell models, other in silico analyses, or animal model studies, and also as potential therapeutic targets in CRC. The role of ADAM8 in KRAS 12/13-mutant CRC tumors, SEMA7A in BRAF-mutant CRC tumors, and ADAMTS10 in PIK3CA-mutated tumors requires further investigation.

## Figures and Tables

**Table 1:** Characteristics of the patient group.

	Female	Male	All
Age	67.5 ± 9.77	65 ± 9.03	66 ± 9.37
Tumor localization			
Left-side tumor	28 (68.29%)	35 (76.09%)	63 (72.41%)
Right-side tumor	13 (31.71%)	11 (23.91%)	24 (27.59%)
T parameter			
T1	0 (0%)	4 (8.7%)	4 (4.6%)
T2	10 (24.39%)	5 (10.87%)	15 (17.24%)
T3	27 (65.85%)	31 (67.39%)	58 (66.66%)
T4	4 (9.76%)	6 (13.04%)	10 (11.49%)
N parameter			
N0	19 (46.34%)	22 (47.83%)	41 (47.12%)
N1	15 (36.59%)	17 (36.96%)	32 (36.78%)
N2	7 (17.07%)	7 (15.22%)	14 (16.1%)
M parameter			
M0	37 (90.24%)	37 (80.43%)	74 (85.06%)
M1	4 (9.76%)	9 (19.57%	13 (14.94%)
TNM Stage			
I	8 (19.51%)	7 (15.22%)	15 (17.24%)
II	10 (24.39%)	13 (28.26%)	23 (26.43%)
III	19 (46.34%)	18 (39.13%)	37 (42.53%)
IV	4 (9.76%)	8 (17.39%)	12 (13.79%)
Grading			
High	4 (9.76%)	8 (17.39%)	12 (13.79%)
Low	37 (90.24%)	38 (82.61%)	75 (86.21%)
MSI Status (*n* = 73)			
MSI-Low	28 (77.78%)	32 (86.49%)	60 (82.19%)
MSI-High	8 (22.22%)	5 (13.51%)	13 (17.81%)
Presurgical treatment			
Yes	5 (12.2%)	6 (13.04%)	11 (12.64%)
No	36 (87.8%)	40 (86.96%)	76 (87.36%)

**Table 2:** KRAS, NRAS, BRAF, PIK3CA, and AKT gene mutation evaluation details.

Gene	Exon	Amino Acid Change	Nucleotide Change	Cosmic ID
KRAS	2	G12A	c.35G>C	522
		G12D	c.35G>A	521
		G12R	c.34G>C	518
		G12C	c.34G>T	516
		G12S	c.34G>A	517
		G12V	c.35G>T	520
		G13D	c.38G>A	532
	3	A59T	c.175G>A	546
		A59E	c.176C>A	547
		A59G	c.176C>G	28518
		Q61H	c.183A>C	554
		Q61H	c.183A>T	555
		Q61L	c.182A>T	553
		Q61R	c.182A>G	552
	4	K117N	c.351A>C	19940
		K117N	c.351A>T	28519
		K117R	c.350A>G	4696722
		K117E	c.349A>G	-
		A146T	c.436G>A	19404
		A146P	c.436G>C	19905
		A146V	c.437C>T	19900
NRAS	2	G12D	c.35G>A	564
		G12S	c.34G>A	563
		G12C	c.34G>T	562
		G13R	c.37G>C	569
		G13V	c.38G>T	574
	3	A59T	c.175G>A	578
		A59D	c.176C>A	253327
		Q61K	c.181C>A	580
		Q61L	c.182A>T	583
		Q61R	c.182A>G	584
		Q61H	c.183A>C	586
		Q61H	c.183A>T	585
	4	K117R	c.350A>G	-
		A146T	c.436G>A	27174
BRAF	15	V600E	c.1799T>A	476
		V600E2	c.1799-1800TG>AA	-
		V600D	c.1799-1800TG>AT	477
		V600K	c.1798-1799GT>AA	473
PIK3CA	9	E542K	c.1624G>A	760
		E545K	c.1633G>A	763
		E545Q	c.1633G>C	27133
	20	H1047R	c.3140A>G	775
		H1047L	c.3140A>T	776
AKT1	4	E17K	c.49G>A	33765

**Table 3:** Cytokines, chemokines, and growth factors sets assigned to the appropriate Gene Ontology (GO) terms and Kyoto Encyclopedia of Genes and Genomes (KEGG) annotations.

Process Name	Cytokines Involved	Origin
Positive regulation of immune system process	MIF, SCF, MCP1, SDF-1a, VEGFA, MCP3, MCSF, MIP-1a, IL-1a, IL-18, IL-6, RANTES, IL-5, TNF-b, LIF, IL-2, IL-1b, IL-7, IFN-g, IL-13, TNF-a, IL-10, IL-8, IL-4, IP-10, IL-15, IL-2Ra, IL-16, CTACK, IL-12p40, MIP-1b, IL-17	GO
Chemokine signaling pathway	IL-8, MCP1, SDF-1a, GRO-a, IP-10, RANTES, MIP-1a, CTACK, Eotaxin, MCP3, MIP-1b	KEGG
JAK-STAT signaling pathway	IL-12p70, IL-12p40, PDGF-bb, GM-CSF, IFN-g, IL-3, IL-5, IL-6, IL-7, IL-8, LIF, IL-9, IL-10, G-CSF, IL-13, IL-15, IL-2Ra, IFN-a2	KEGG
MAPK signaling pathway	bNGF, IL-1b, PDGF-bb, IL-1a, BasicFGF, SCF, MCSF, TNF-a, HGF, VEGFA	KEGG
Interleukin-10 signaling	MCP1, MCSF, IL-8, IL-18, IL-6, GM-CSF, LIF, IL-10, IL-1Ra, IL-1a, IP-10, GRO-a, MIP-1a, IL-1b, MIP-1b, G-CSF, RANTES, TNF-a	KEGG
Toll-like signaling pathway	IL-8, TNF-a, IL-6, IP-10, RANTES, IL-1b, MIP-1b, MIG, IFN-a2	KEGG
Regulation of cell population proliferation	CTACK, PDGF-bb, LIF, SCF, MIF, BasicFGF, IFN-g, IL-4, GM-CSF, G-CSF, IL-7, IL-3, MCSF, SDF-1a, SCGF-b, IL-2Ra, TNF-a, IL-6, IL-1b, IL-1a, IP-10, RANTES, IL-5, Eotaxin, IL-2Ra, IL-10, IL-2, IL-18, IL-15, IL-13, IL-9, TNF-b)	KEGG
Leukocyte activation	IL-4, IL-15, IFN-g, SCF, IL-2Ra, IL-8, MCSF, IL-13, IL-18, MIP-1a, RANTES, IL-10, GM-CSF, IL-9, IL-7, IFN-a2, IL-2, IL-6, TNF-a	GO
Inflammatory response	IL-9, CTACK, Eotaxin, MCP1, IFN-a2, IL-1Ra, IL-2Ra, IFN-g, IL-15, IL-1a, IL-6, IL-17, IL-4, MCP3, MIP-1a, IL-18, CTACK, MIF, TNF-a, RANTES, MCSF, MIG, IL-1b, IL-5, IL-10, IL-8, IL-13, IP-10, MIP-1b	GO
Positive regulation of cytokine production	IL-9, IL-12p70, GM-CSF, IL-10, HGF, IL-2, IL-15, IL-1b, IL-18, IFN-g, IL-7, IL-4, TNF-a, IL-17, MIF, TNF-b, IL-16, IL-13, MIP-1a, IL-1a, IL-6	GO

MIF—Macrophage Migration Inhibitory Factor, SCF—Stem Cell Factor, MCP1—Monocyte Chemoattractant Protein 1, SDF-1a—Stromal Cell-Derived Factor 1 alpha, VEGFA—Vascular Endothelial Growth Factor A, MCP3—Monocyte Chemoattractant Protein 3, MCSF—Macrophage Colony-Stimulating Factor, MIP-1a—Macrophage Inflammatory Protein 1 alpha, IL-1a—Interleukin 1 alpha, IL-18—Interleukin 18, IL-6—Interleukin 6, RANTES—Regulated on Activation, Normal T Cell Expressed and Secreted, IL-5—Interleukin 5, TNF-b—Tumor Necrosis Factor beta, LIF—Leukemia Inhibitory Factor, IL-2—Interleukin 2, IL-1b—Interleukin 1 beta, IL-7—Interleukin 7, IFN-g—Interferon gamma, IL-13—Interleukin 13, TNF-a—Tumor Necrosis Factor alpha, IL-10—Interleukin 10, IL-8—Interleukin 8, IL-4—Interleukin 4, IP-10—Interferon gamma-induced protein 10, IL-15—Interleukin 15, IL-2Ra—Interleukin 2 Receptor alpha, IL-16—Interleukin 16, CTACK—Cutaneous T Cell-Attracting Chemokine, IL-12p40—Interleukin 12 subunit p40, MIP-1b—Macrophage Inflammatory Protein 1 beta, IL-17—Interleukin 17, GRO-a—Growth-regulated alpha protein, IL-12p70—Interleukin 12 subunit p70, PDGF-bb—Platelet-Derived Growth Factor BB, GM-CSF—Granulocyte-Macrophage Colony-Stimulating Factor, IL-3—Interleukin 3, G-CSF—Granulocyte Colony-Stimulating Factor, IFN-a2—interferon-alpha 2, bNGF—beta-Nerve Growth Factor, MIG—Monokine induced by gamma interferon, HGF—Hepatocyte Growth Factor, BasicFGF—Basic Fibroblast Growth Factor, SCGF-b—Stem Cell Growth Factor beta, IL-1Ra—Interleukin 1 Receptor antagonist.

**Table 4:** SEMA7A, SEMA4D, ADAM8, and ADAMTS10 protein concentration and TNM scale parameters, and tumor stage. T, N, and tumor stage *p*-value and tau were obtained from Kendall’s Tau rank correlation coefficient; *p*-value for M parameter—from U-Mann–Whitney test.

	T Parameter		N Parameter		M Parameter	Tumor Stage	
	*p*-Value	tau	*p*-Value	tau	*p*-Value	*p*-Value	tau
SEMA7A	0.2919	−0.09008	0.6822	0.03498	0.3969	0.6161	0.04151
SEMA4D	0.5348	0.05295	0.7577	−0.02631	0.5554	0.786	0.02244
ADAM8	0.8257	−0.01814	0.9189	−0.008337	0.5402	0.45	−0.05988
ADAMTS10	0.2305	−0.09912	0.7435	−0.02688	0.6401	0.9748	−0.002515

**Table 5:** SEMA7A, SEMA4D, ADAM8, and ADAMTS10 expression based on grading, primary tumor location (left-sided vs. right-sided), and microsatellite instability (MSI) status. U-Mann–Whitney test.

	Grading *p*-Value	Localization *p*-Value	MSI Status *p*-Value
SEMA7A	0.846	0.797	0.795
SEMA4D	0.393	0.298	0.316
ADAM8	0.662	0.8923	0.5174
ADAMTS10	0.2368	0.3281	0.2727

**Table 6:** KRAS, NRAS, PIK3CA, BRAF, and AKT mutation occurrence in cohort of 54 CRC tumors.

Gene	Mutation Status		Percent (%)	
	Wild-Type	Mutant	Percent among Mutation of the One Gene	Percent among All Group
KRAS	36	18		33.33%
KRAS-117-STATUS	51	3	16.67%	5.55%
KRAS-12/13-STATUS	43	11	61.11%	20.37%
KRAS-59-STATUS	51	3	16.67%	5.55%
KRAS-146-STATUS	52	2	11.11%	3.70%
KRAS-61-STATUS	52	2	11.11%	3.70%
NRAS	44	10		18.52%
NRAS-12-13-STATUS	49	5	50%	9.26%
NRAS-61-STATUS	49	5	50%	9.26%
PIK3CA	50	4		7.41%
PIK3CA 542/545	51	3	75%	5.55%
PIK3CA 1047	53	1	25%	1.85%
BRAF	50	4		7.41%
AKT	53	1		1.85%

**Table 7:** Eigenvalue and the percentage of explained variance for 3 factors (principal components) from the PCA for terms associated with SEMA4D protein expression.

Factor	Eigenvalue	Variance (%)	Cumulative Variance (%)
Toll-like signaling pathway			
Factor 1	4.89295722	54.3661913	54.36619
Factor 2	1.18176034	13.1306704	67.49686
Factor 3	0.96057685	10.6730761	78.16994
Inflammatory response			
Factor 1	13.37143	47.75509	47.75509
Factor 2	4.061744	14.50623	62.26132
Factor 3	2.513067	8.975241	71.23656
Leukocyte activation			
Factor 1	10.147664342	53.408759696	53.40876
Factor 2	2.497374683	13.144077281	66.55284
Factor 3	1.942422870	10.223278263	76.77612

**Table 8:** Eigenvalue and the percentage of explained variance for 3 factors (principal components) from the PCA for terms associated with SEMA7A protein expression.

Factor	Eigenvalue	Variance (%)	Cumulative Variance (%)
JAK-STAT signaling pathway			
Factor 1	9.693638072	53.85354485	53.85354
Factor 2	2.599087884	14.43937713	68.29292
Factor 3	1.711508394	9.50837996	77.80130
Interleukin-10 signaling			
Factor 1	8.549978302	47.49987945	47.49988
Factor 2	2.776434574	15.42463652	62.92452
Factor 3	1.640473900	9.11374389	72.03826
MAPK signaling pathway			
Factor 1	4.45896812	44.5896812	44.58968
Factor 2	2.35012995	23.5012995	68.09098
Factor 3	1.08965639	10.8965639	78.98754

## Data Availability

GSEA data: Dampier CH (2020). “FieldEffectCrc: Tumor, tumor-adjacent normal, and healthy colorectal transcriptomes as SummarizedExperiment objects”. https://bioconductor.org/packages/release/data/experiment/html/FieldEffectCrc.html (accessed on 15 November 2022). Experimental data can be shared up on request.

## Supplementary Materials

The following supporting information can be downloaded at: https://www.mdpi.com/article/10.3390/cimb46090609/s1, Figure S1: SEMA7A and ADAM8 correlation. Spearman’s rank correlation; Figure S2: SEMA7A and ADAMTS10 correlation. Spearman’s rank correlation; Figure S3: SEMA7A and SEMA4D correlation. Spearman’s rank correlation; Figure S4: SEMA4D and ADAMTS10 correlation. Spearman’s rank correlation; Table S1: Loadings of 3 factors (PCA Factors) after varimax rotation. Coordinates for the variables for molecular processes associated with SEMA4D protein expression in PCA; Figure S5: Correlation between SEMA4D protein expression and Toll-like signaling pathway PCA Factor 1. Spearman’s rank correlation; Figure S6: Correlation between SEMA4D protein expression and Inflammatory Response PCA Factor 1. Spearman’s rank correlation; Figure S7: Correlation between SEMA4D protein expression and Leukocyte Activation PCA Factor 1. Spearman’s rank correlation; Table S2: Loadings of 3 factors (PCA Factors) after varimax rotation. Coordinates for the variables for molecular processes associated with SEMA7A protein expression in PCA; Figure S8: Correlation between SEMA7A protein expression and JAK-STAT Signaling Pathway PCA Factor 1. Spearman’s rank correlation; Figure S9: Correlation between SEMA7A protein expression and IL-10 Signaling PCA Factor 3. Spearman’s rank correlation; Figure S10: Correlation between SEMA7A protein expression and MAPK Signaling Pathway PCA Factor 3. Spearman’s rank correlation.
