# Supplementary material for: Associations of SEMA7A, SEMA4D, ADAMTS10, and ADAM8 with KRAS, NRAS, BRAF, PIK3CA, and AKT Gene Mutations, Microsatellite Instability Status, and Cytokine Expression in Colorectal Cancer Tissue
Source: Curr Issues Mol Biol. 2024 Sep 15;46(9):10218–48. doi: 10.3390/cimb46090609 (PMC11431007; doi:10.3390/cimb46090609)
Supplement: Supplementary file 1 [file cimb-46-00609-s001.zip › cimb-3199276-supplementary.pdf]

current issues in molecular biology

MDPI

Article

Associations of SEMA7A, SEMA4D, ADAMTS10, and ADAM8 with KRAS, NRAS, BRAF, PIK3CA, and AKT Gene Mutations, Microsatellite Instability Status, and Cytokine Expression in Colorectal Cancer Tissue

Błażej Ochman [1], Piotr Limanówka [1], Sylwia Mielcarska [1], Agnieszka Kula [2], Miriam Dawidowicz [2], Wiktor Wagner [1], Dorota Hudy [1], Monika Szrot [3], Jerzy Zbigniew Piecuch [3], Jerzy Piecuch [3], Zenon Czuba [4] and Elżbieta Świętochowska [1,*]

1 Department of Medical and Molecular Biology, Faculty of Medical Sciences in Zabrze, Medical University ofSilesia, 19 Jordana, 41-808 Zabrze, Poland;
2 Department of Oncological Surgery, Faculty of Medical Sciences in Zabrze, Medical University of Silesia, 41-808 Katowice, Poland;
3 Department of General and Bariatric Surgery and Emergency Medicine in Zabrze, Faculty of Medical Sciences in Zabrze, Medical University of Silesia, 10 Marii Curie-Skłodowskiej, 41-800 Zabrze, Poland;
4 Department of Microbiology and Immunology, Faculty of Medical Sciences in Zabrze, Medical University of Silesia, 19 Jordana, 41-808 Zabrze, Poland
* Correspondence: eswietochowska@sum.edu.pl

Supplementary Material

Citation: Ochman, B.; Limanówka, P.; Mielcarska, S.; Kula, A.; Dawidowicz, M.; Wagner, W.; Hudy, D.; Szrot, M.; Piecuch, J.Z.; Piecuch, J.; et al. Associations of SEMA7A, SEMA4D, ADAMTS10, and ADAM8 with KRAS, NRAS, BRAF, PIK3CA, and AKT Gene Mutations, Microsatellite Instability Status, and Cytokine Expression in Colorectal Cancer Tissue. *Curr. Issues Mol. Biol.* **2024**, *46*, 10218–10248. ttps://doi.org/10.3390/cimb46090609.

Academic Editor: Liudmila V. Spirina, Irina V. Kondakova and Natalya V. Yunusova

Received: 25 August 2024
Revised: 11 September 2024
Accepted: 13 September 2024
Published: 15 September 2024

Copyright: © 2024 by the authors. Submitted for possible open access publication under the terms and conditions of the Creative Commons Attribution (CC BY) license (https://creativecommons.org/licenses/by/4.0/).

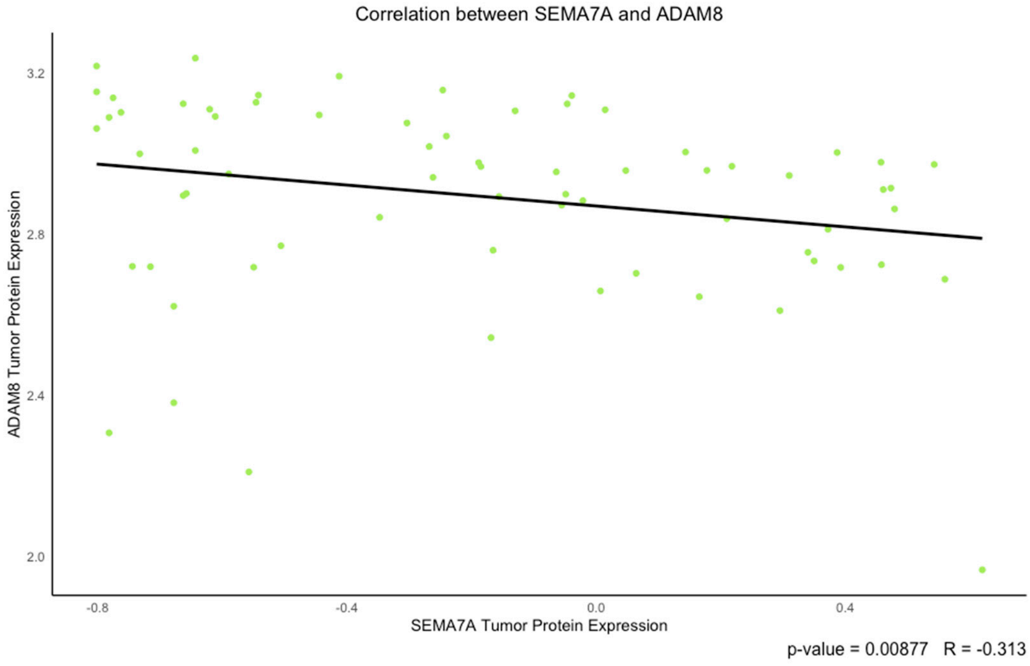

Figure S1. SEMA7A and ADAM8 correlation. Spearman's rank correlation.

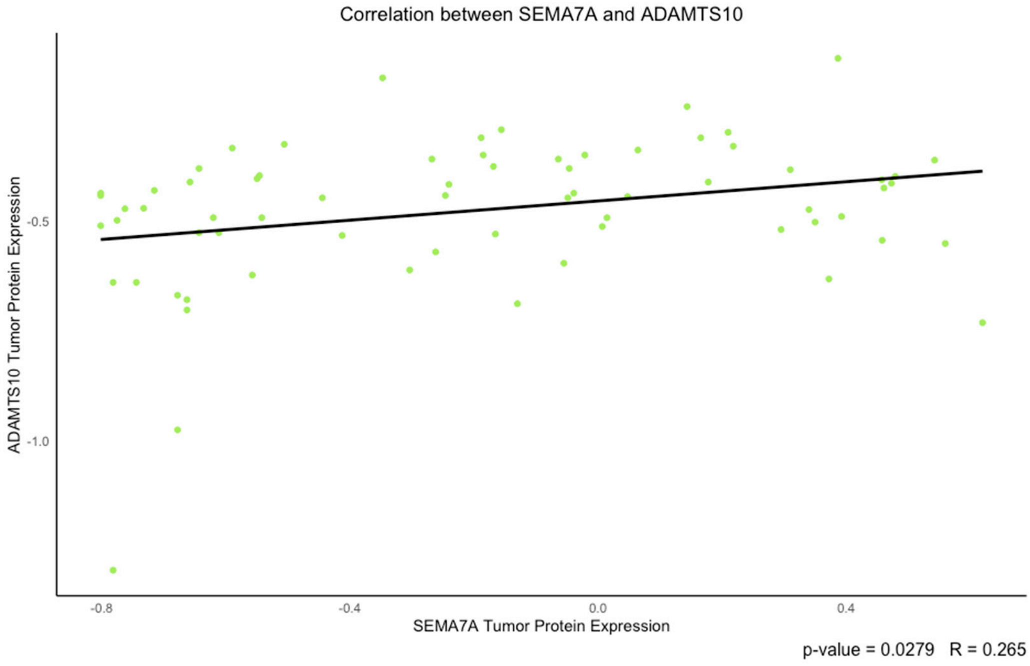

Figure S2. SEMA7A and ADAMTS10 correlation. Spearman's rank correlation.

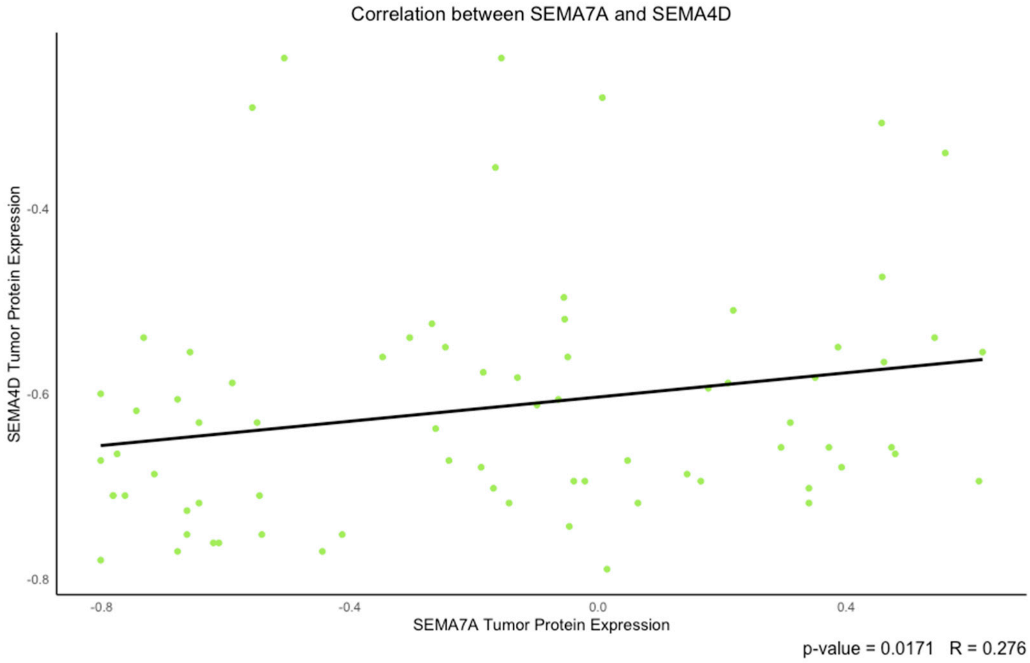

Figure S3. SEMA7A and SEMA4D correlation. Spearman's rank correlation.

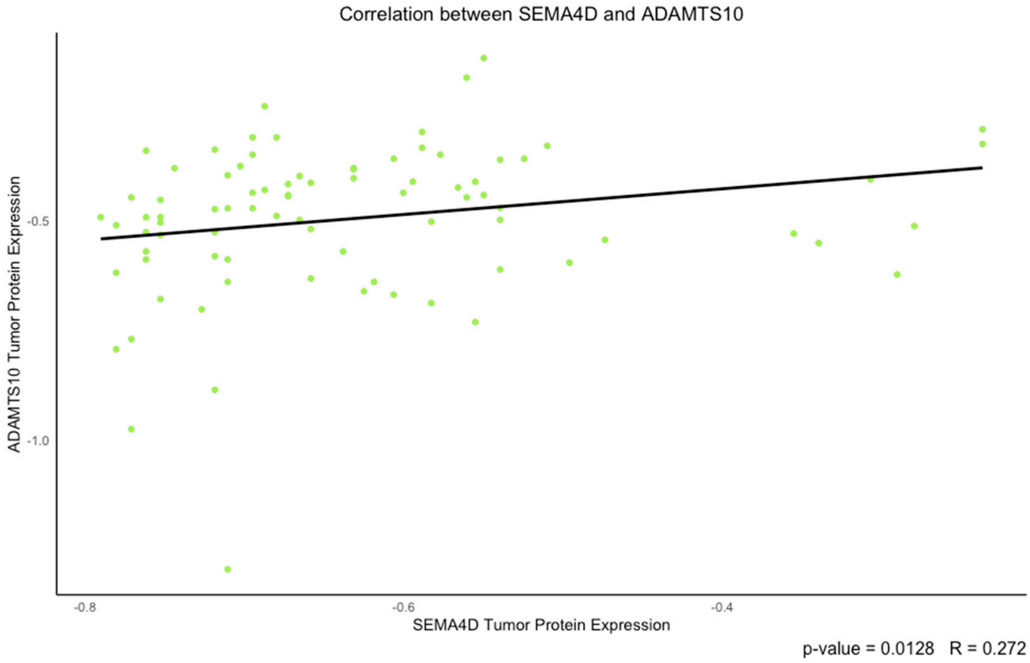

Figure S4. SEMA4D and ADAMTS10 correlation. Spearman's rank correlation.

Table S1. Loadings of 3 factors (PCA Factors) after varimax rotation. Coordinates for the variables for molecular processes associated with SEMA4D protein expression in PCA.

Variable	Factor 1	Factor 2	Factor 3
Toll-like signaling pathway			
IL-8	0.8654129	0.12082868	−0.27832677
TNF-a	0.7646254	−0.57456393	0.04243603
IL-6	0.5520321	−0.38637508	−0.08314433
IP-10	0.7412093	0.24245448	0.34048522
RANTES	0.8876157	0.24209875	0.30374570
IL-1b	0.5088703	0.03359059	−0.78865237
MIP-1b	0.7329184	0.21100264	0.10733257
MIG	0.7464454	0.50145339	−0.08627164
IFN-a2	0.7510489	−0.52273266	0.15896540
Inflammatory Response			
IL-9	0.908600937	−0.32890339	−0.05068055
CTACK	0.894026864	0.10838820	−0.35198564
Eotaxin	0.819986873	0.08900517	0.03945236
MCP1	0.733748228	−0.49996173	0.03181336
IFN-a2	0.901154942	0.22190152	−0.23097914
IL-1Ra	0.488805956	0.15149237	0.34305817
IL-2Ra	0.853938809	0.07262477	0.05459024
IFN-g	0.447744313	0.48743094	0.38113586
IL-15	0.718845504	0.42286881	−0.29808614
IL-1a	0.634089609	0.53494935	0.18249841
IL-6	0.526102401	−0.20455367	−0.24986346
IL-17	0.583594480	0.11928565	0.08824774
IL-4	0.861614182	0.19077054	−0.20109329
MCP3	0.821075885	0.27098042	−0.33391342
MIP-1a	0.588915633	−0.71512131	−0.17401599
IL-18	0.800331600	−0.30413510	−0.02818448
MIF	0.006016992	−0.60887398	−0.01495696
TNF-a	0.904220312	0.24968806	−0.18887330
RANTES	0.768333158	−0.53175078	0.10603131
MCSF	0.669870971	0.27323380	0.53931679
MIG	0.551769594	−0.46071326	0.47585030
IL-1b	0.438269254	0.28125183	0.69969458
IL-5	0.406832362	0.71071423	−0.10909693
IL-10	0.791540601	0.26970252	−0.33201866
IL-8	0.762483782	−0.17913175	0.39990466
IL-13	0.286289606	−0.27090886	−0.55120846
IP-10	0.678824345	−0.39904962	0.14902050
MIP-1b	0.587888518	−0.45790762	0.16507643
Leukocyte Activation			
IL-4	0.8621145	−0.21063257	−0.01619217
IL-15	0.7528651	−0.39217814	−0.26374188
IFN-g	0.3869067	−0.52466834	−0.01792524
SCF	0.7029633	0.36661631	−0.25052082
IL-2Ra	0.8347054	−0.24723877	0.21956046
IL-8	0.6836284	−0.07278409	0.56909430

**Variable**	**Factor 1**	**Factor 2**	**Factor 3**
MCSF	0.5556702	−0.51197340	0.42794971
IL-13	0.4037088	0.61653253	−0.42153697
IL-18	0.8148456	0.22314262	0.28010760
MIP-1a	0.5918515	0.68031527	0.29531769
RANTES	0.7047315	0.30510383	0.54001070
IL-10	0.8513180	−0.04877440	−0.35578514
GM-CSF	0.7018462	−0.09616087	−0.23105580
IL-9	0.8808144	0.21898956	0.28637747
IL-7	0.6601777	0.37779854	−0.39166412
IFN-a2	0.9193341	−0.21530767	−0.08709482
IL-2	0.7414459	−0.39286735	−0.41610803
IL-6	0.6044558	0.37808422	−0.09276581
TNF-a	0.9261431	−0.17086013	−0.12085306

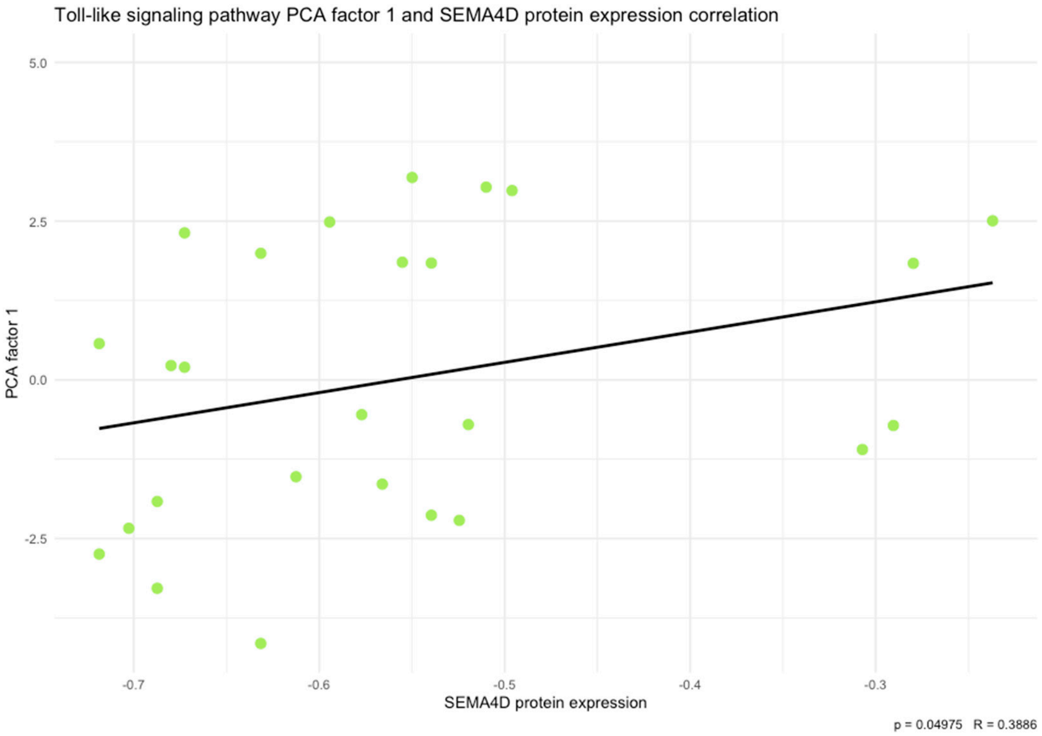

Figure S5. Correlation between SEMA4D protein expression and Toll-like signaling pathway PCA Factor 1. Spearman's rank correlation.

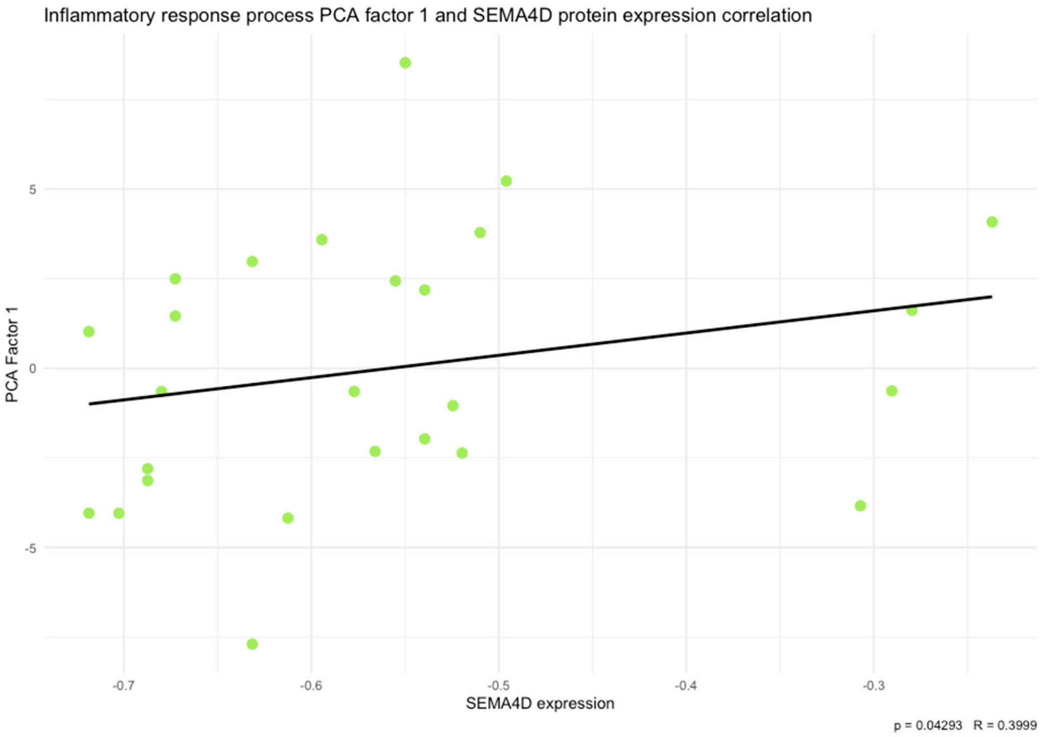

Figure S6. Correlation between SEMA4D protein expression and Inflammatory Response PCA Factor 1. Spearman's rank correlation.

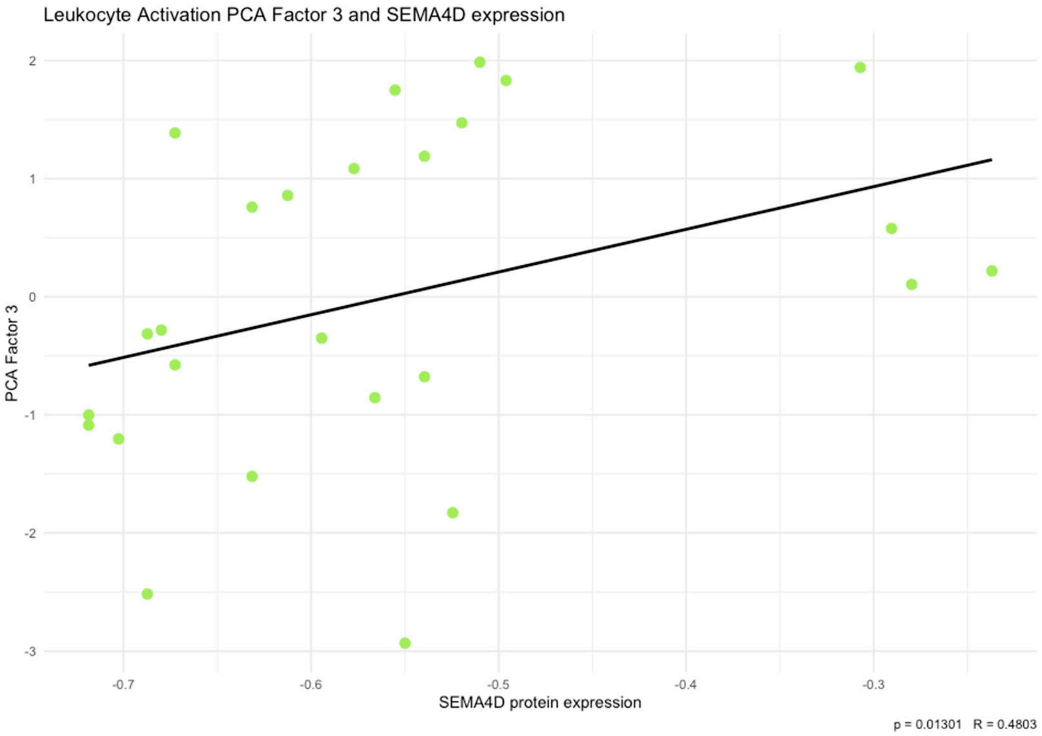

Figure S7. Correlation between SEMA4D protein expression and Leukocyte Activation PCA Factor 1. Spearman's rank correlation.

Table S2. Loadings of 3 factors (PCA Factors) after varimax rotation. Coordinates for the variables for molecular processes associated with SEMA7A protein expression in PCA.

	**Factor 1**	**Factor 2**	**Factor 3**
JAK-STAT Signaling pathway			
IL-12p70	−0.8258624	0.11750205	−0.457282328
IL-12p40	−0.8373085	0.22487476	−0.273070868
PDGF-bb	−0.8150474	−0.35654009	0.293391183
GM-CSF	−0.7318521	−0.09920407	−0.009131147
IFN-g	−0.4345574	−0.38886051	0.005565871
IL-3	−0.9030440	−0.22457675	−0.168425370
IL-5	−0.5419631	−0.59561754	−0.440889888
IL-6	−0.5894099	0.36691639	0.281352065
IL-7	−0.6578941	0.56600596	−0.212041374
IL-8	−0.6088077	−0.15935003	0.677005337
LIF	−0.6822913	−0.36118515	0.300599048
IL-9	−0.7968022	0.24426849	0.333012959
IL-10	−0.9025569	0.12695732	−0.256924516
G-CSF	−0.5905676	0.69535976	0.275303348
IL-13	−0.4074818	0.74137612	−0.133061854
IL-15	−0.8195435	−0.25087146	−0.275605837
IL-2Ra	−0.8228323	−0.20592529	0.310738099
IFN-a2	−0.9397476	−0.12733614	−0.056136555
Interleukin-10 Signaling			
MCP1	0.7943372	−0.3767467	0.02166584
MCSF	0.6362290	0.5551807	0.15726514
IL-8	0.8170250	0.1309014	0.05806426
IL-18	0.8552311	−0.1131509	−0.15993176
IL-6	0.5969671	−0.1126385	−0.13581595
GM-CSF	0.5455173	0.1217519	−0.58076828
LIF	0.6765232	0.4198803	−0.22605400
IL-10	0.7198566	0.1825030	−0.07950679
IL-1Ra	0.4971078	0.2547767	0.68842510
IL-1a	0.5637802	0.6349949	0.20255925
IP-10	0.7202554	−0.2241361	0.01494021
GRO-a	0.5420590	0.1601852	−0.62518396
MIP-1a	0.7035367	−0.6550089	0.11742772
IL-1b	0.4579680	0.6674594	0.27835836
MIP-1b	0.6971988	−0.3087812	0.35561877
G-CSF	0.7320699	−0.5831195	0.12585389
RANTES	0.8335233	−0.3698583	0.05552647
TNF-a	0.8284932	0.2616883	−0.17454436
MAPK Signaling Pathway			
bNGF	0.7650901	−0.32585012	−0.18556043
IL-1b	0.6077804	0.61540223	0.26322338
PDGF-bb	0.8447211	0.01907708	−0.32578568
IL-1a	0.8095839	0.05862270	0.24379058
BasicFGF	0.3503633	0.50349412	−0.70831476
SCF	0.4540817	−0.71927173	−0.07608305
MCSF	0.8046069	0.44958270	0.07520154
TNF-a	0.8924766	−0.24856940	−0.02908210
HGF	0.1149185	−0.90042150	−0.10967790
VEGFA	0.5909068	−0.12610632	0.54252954

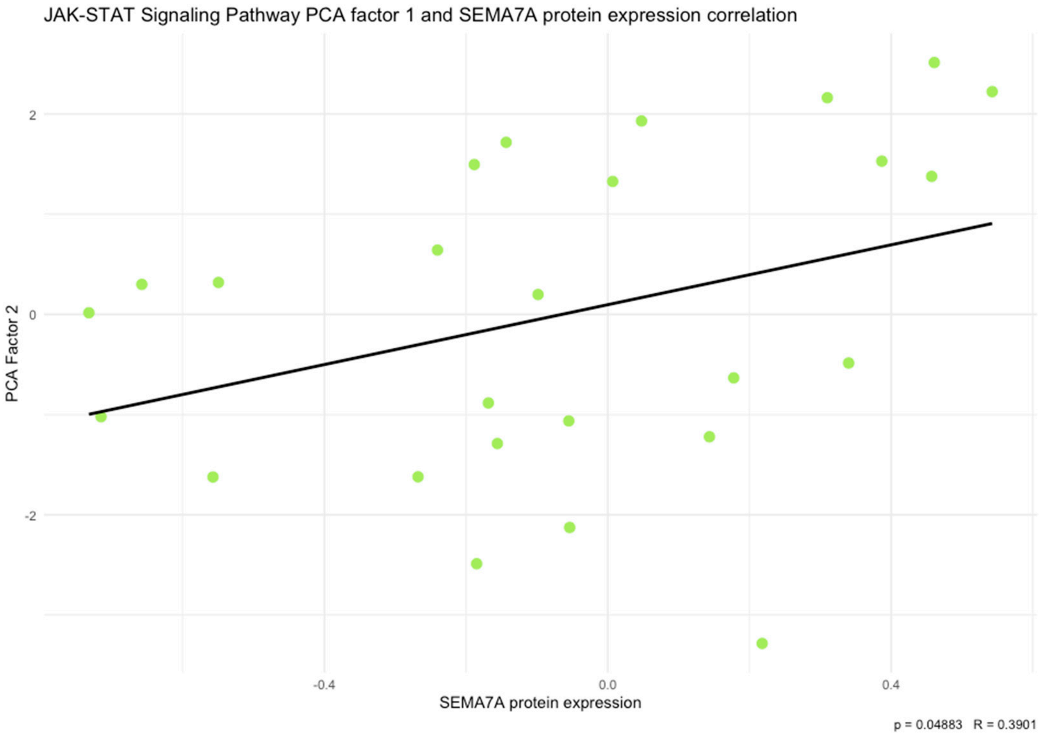

Figure S8. Correlation between SEMA7A protein expression and JAK-STAT Signaling Pathway PCA Factor 1. Spearman's rank correlation.

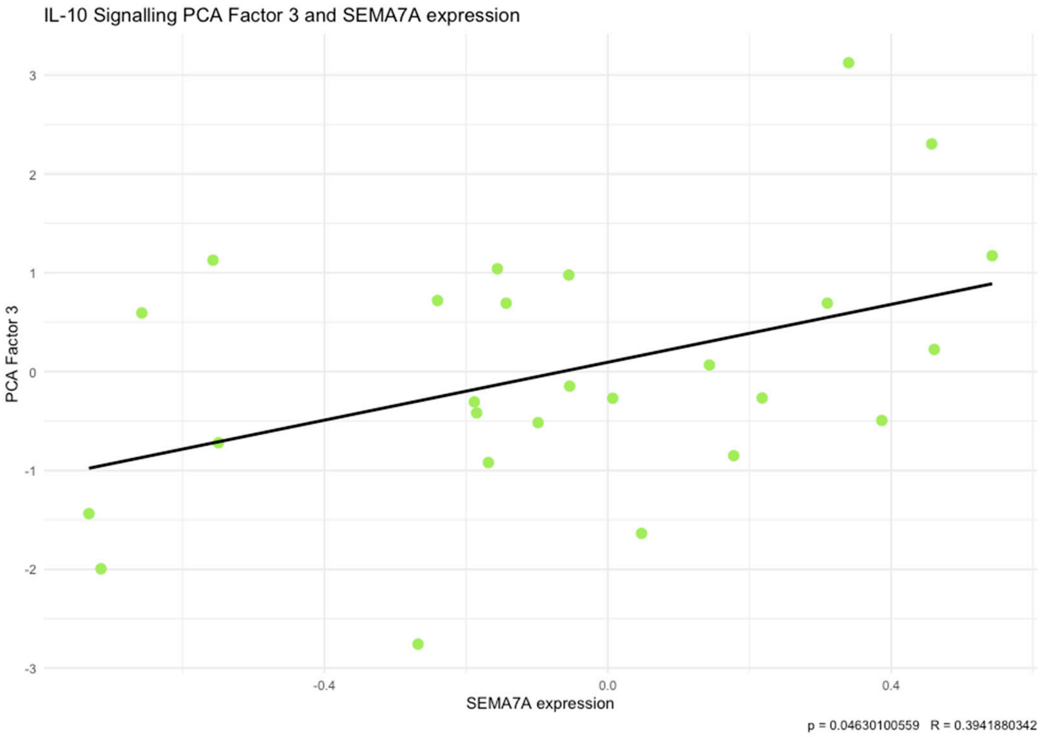

Figure S9. Correlation between SEMA7A protein expression and IL-10 Signaling PCA Factor 3. Spearman's rank correlation.

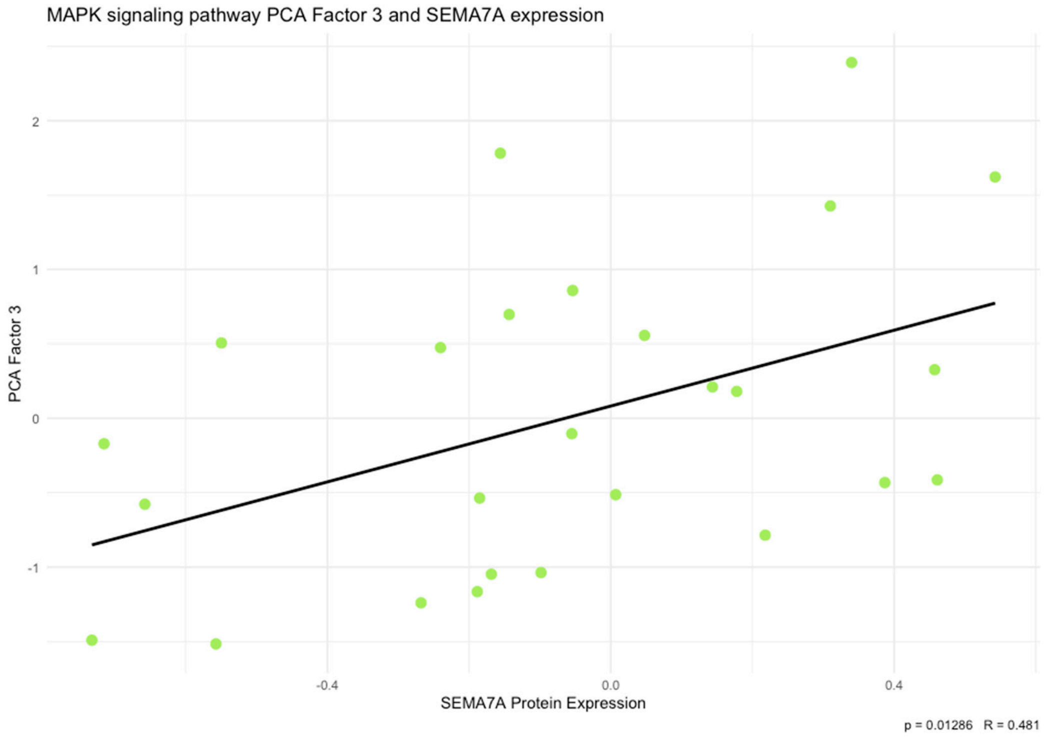

Figure S10. Correlation between SEMA7A protein expression and MAPK Signaling Pathway PCA Factor 3. Spearman’s rank correlation.
